# Hyperinsulinaemic–hypoglycaemic glucose clamps in human research: a systematic review of the literature

**DOI:** 10.1007/s00125-020-05361-8

**Published:** 2021-02-10

**Authors:** Therese W. Fabricius, Clementine E. M. Verhulst, Peter L. Kristensen, Cees J. Tack, Rory J. McCrimmon, Simon Heller, Mark L. Evans, Stephanie A. Amiel, Thomas R. Pieber, Bastiaan E. de Galan, Ulrik Pedersen-Bjergaard, on behalf of the Hypo-RESOLVE consortium

**Affiliations:** 1 Department of Endocrinology and Nephrology, Nordsjællands Hospital, Hillerød, Denmark; 2 Department of Internal Medicine, Radboud University Medical Centre, Nijmegen, the Netherlands; 3 Department of Internal Medicine, University of Dundee, Dundee, UK; 4 Department of Oncology and Metabolism, University of Sheffield, Sheffield, UK; 5 Wellcome Trust/MRC Institute of Metabolic Science, University of Cambridge, Cambridge, UK; 6 Department of Diabetes, School of Life Course Sciences, Faculty of Life Sciences & Medicine, King’s College London, London, UK; 7 Division of Endocrinology and Diabetology, Department of Internal Medicine, Medical University of Graz, Graz, Austria; 8 Department of Internal Medicine, Maastricht University Medical Centre, Maastricht, the Netherlands; 9 Department of Clinical Medicine, Faculty of Health and Medical Sciences, University of Copenhagen, Copenhagen, Denmark

**Keywords:** Diabetes, Diabetes mellitus, Human, Hyperinsulinaemic–hypoglycaemic clamp, Hypoglycaemia, Systematic review, Type 1 diabetes, Type 2 diabetes

## Abstract

**Aims/hypothesis:**

The hyperinsulinaemic–hypoglycaemic glucose clamp technique has been developed and applied to assess effects of and responses to hypoglycaemia under standardised conditions. However, the degree to which the methodology of clamp studies is standardised is unclear. This systematic review examines how hyperinsulinaemic–hypoglycaemic clamps have been performed and elucidates potential important differences.

**Methods:**

A literature search in PubMed and EMBASE was conducted. Articles in English published between 1980 and 2018, involving adults with or without diabetes, were included.

**Results:**

A total of 383 articles were included. There was considerable variation in essential methodology of the hypoglycaemic clamp procedures, including the insulin dose used (49-fold difference between the lowest and the highest rate), the number of hypoglycaemic steps (range 1−6), the hypoglycaemic nadirs (range 2.0–4.3 mmol/l) and the duration (ranging from 5 to 660 min). Twenty-seven per cent of the articles reported whole blood glucose levels, most venous levels. In 70.8% of the studies, a dorsal hand vein was used for blood sampling, with some form of hand warming to arterialise venous blood in 78.8% of these. Key information was missing in 61.9% of the articles.

**Conclusions/interpretation:**

Although the hyperinsulinaemic–hypoglycaemic clamp procedure is considered the gold standard to study experimental hypoglycaemia, a uniform standard with key elements on how to perform these experiments is lacking. Methodological differences should be considered when comparing results between hypoglycaemic clamp studies.

**PROSPERO registration:**

This systematic review is registered in PROSPERO (CRD42019120083).

**Graphical abstract:**

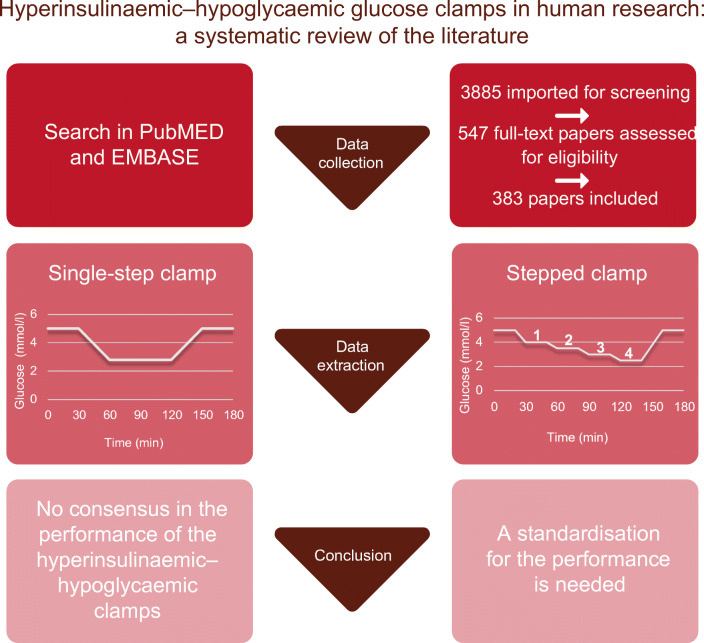

**Supplementary Information:**

The online version contains peer-reviewed but unedited supplementary material available at 10.1007/s00125-020-05361-8.

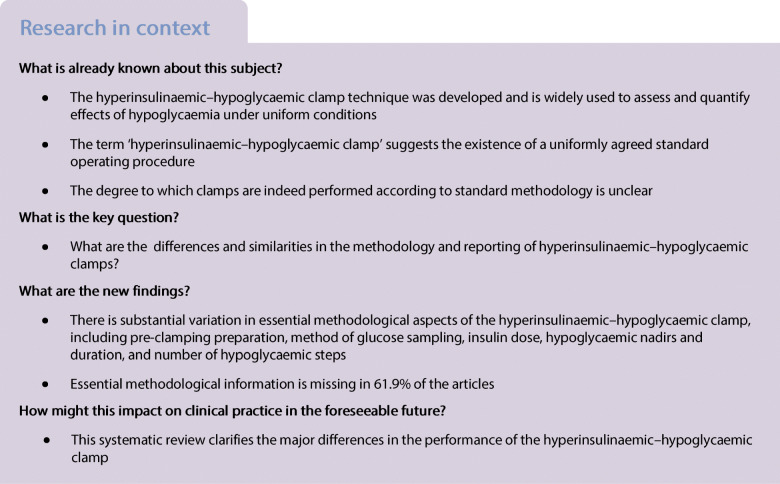



## Introduction

Despite important advances in the manufacturing of insulin agents, insulin administration and glucose monitoring, hypoglycaemia remains the most frequent adverse event in people with diabetes treated with insulin and is associated with increased morbidity and mortality [1]. Revealing the still many knowledge gaps in understanding the pathophysiology of, responses to and effects of hypoglycaemia is therefore important.

In the 1970s, the hyperinsulinaemic–normoglycaemic clamp technique was developed for quantification of beta cell sensitivity to glucose and of tissue sensitivity to insulin [2, 3]. The hyperinsulinaemic–hypoglycaemic clamp technique is a variant of this method, designed to assess clinical manifestations of hypoglycaemia, including counterregulatory hormone responses, symptomatic awareness and cognitive function, under standardised conditions [4, 5]. It consists of continuous intravenous insulin infusion at a (relatively) high dose to ensure sufficient glucose lowering and a variable infusion of glucose guided by glucose measurements performed at regular time intervals to achieve stable glucose values at pre-defined target(s). Two forms can be distinguished, involving either a single glucose target (single-step clamp) or multiple decremental targets in the hypoglycaemic range (stepped clamp).

A universally accepted standardised protocol for the hyperinsulinaemic–hypoglycaemic clamp would allow for meta-analysis with increased statistical power when different studies are compared. However, to the best of our knowledge, no previous article has been published that sets out standards for performing hypoglycaemic clamp experiments, for instance with respect to the optimal glucose target or duration of the clamp. Differences in its execution may affect the validity when data from clamp studies are compared or render it impossible to compare study results. In this review, we provide a comprehensive overview on how hyperinsulinaemic–hypoglycaemic clamps have been performed in humans and elucidate differences and similarities in their execution.

## Methods

We performed a descriptive systematic review in accordance with the published protocol in PROSPERO (https://www.crd.york.ac.uk/prospero/display_record.php?RecordID=120083). All peer-reviewed articles available online reporting hyperinsulinaemic–hypoglycaemic clamps involving adults with type 1 diabetes or type 2 diabetes or without diabetes (healthy participants) were included. Only English language articles were included in this review. All articles from studies in which participants had undergone a hyperinsulinaemic–hypoglycaemic clamp were read. If multiple articles were published from the same study, only the first published article with a sufficient clamp description was included, and the hyperinsulinaemic–hypoglycaemic clamp needed to be the main method of the study. Studies involving animals or with inadequate descriptions on how the clamp was performed were excluded. Reporting is in accordance with the Preferred Reporting Items for Systematic Reviews and Meta-Analyses (PRISMA) guidelines [6].

### Data sources and search strategy

A literature search was conducted in PubMed and EMBASE in November 2018. All articles available online in the databases were included, resulting in the inclusion of articles from 1980 to 2018. The search used a combination of free text words and MeSH (PubMed) and Emtree (EMBASE) terms. All titles and abstracts identified from the electronic search via PubMed and EMBASE were imported to COVIDENCE software, version 1.0 (https://www.covidence.org/), accessed in November 2018, a program that streamlines the review process. The search strategy was developed in collaboration with an information specialist at Nordsjællands Hospital with input from clinicians and academics in the review team. The search strategy for PubMed is available in the supplementary material (electronic supplementary material [ESM] Methods).

### Study selection

Duplicates from the two searches were automatically removed when imported into COVIDENCE, version 1.0 (https://www.covidence.org/), accessed in November 2018. All titles and abstracts were assessed independently to identify articles requiring full-text review against the inclusion and exclusion criteria. This first step was done by one reviewer (T.W. Fabricius), using the words ‘clamp’ and ‘hypoglycaemia’. Eligible articles identified after title and abstract review underwent all full-text reading, and the reference lists were searched for other articles. This step was carried out by two reviewers (T.W. Fabricius and C.E.M. Verhulst). For this review we focused on type 1 diabetes, type 2 diabetes and people without diabetes; studies in children were included. Studies exclusively performed in patients with other conditions were excluded (ESM Methods)*.* An extraction sheet was used to extract the desired information from each article. Any disagreements between the reviewers were resolved by consensus and in consultation with one of the senior authors (U. Pedersen-Bjergaard and B.E. de Galan). The list of the included articles is shown in the supplementary material (ESM Methods, ESM Table 1).

### Data extraction

We extracted information from the articles with our main focus on the procedure and the quality of the clamp, including duration of the clamp, number of glucose steps, glucose levels targeted and achieved, duration of target glucose levels, type of insulin and insulin infusion rates (IIRs) used, source of blood sampling (venous, arterial, capillary; arterialisation method in the case of venous blood) and type of glucose analyser used. Furthermore, we collected study characteristics such as author identification, year of publication, type of study and characteristics of the study population.

### Statistics

Results are shown with descriptive statistical methods. We report the continuous data as means with standard deviations in the case of normal distribution, and as medians with interquartile ranges when data are not normally distributed. Statistical analyses were performed using IBM SPSS Statistics version 25. A *p* value <0.05 was considered statistically significant.

## Results

**Fig. 1:**
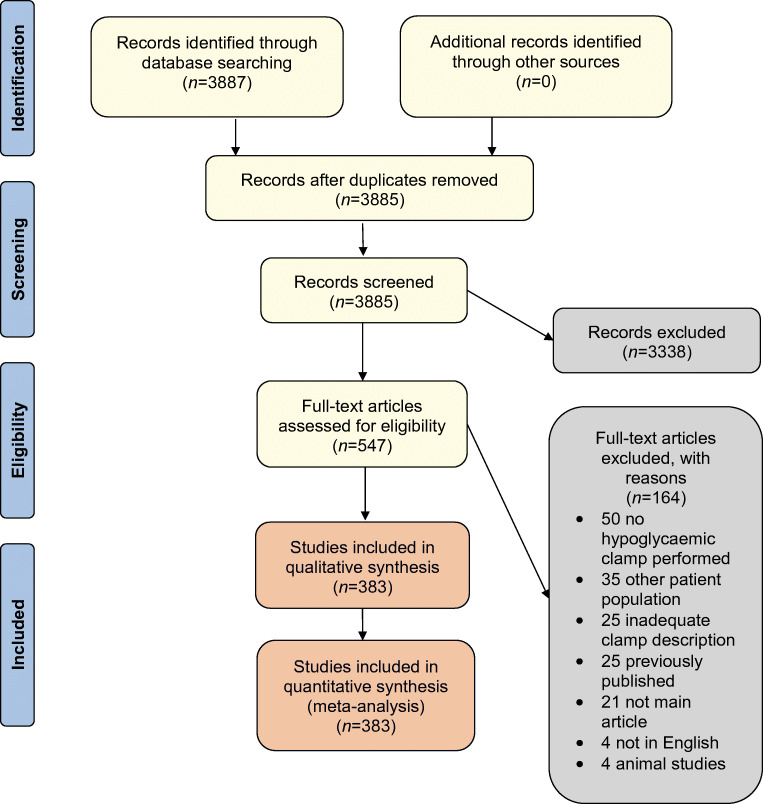
PRISMA flow diagram

A total of 3885 articles were found on PubMed and EMBASE (Fig. 1). After the process of screening, 408 articles fulfilled the inclusion criteria, after which 25 articles were excluded because of inadequate clamp description. A total of 383 articles were thus included for analysis. In total, 38.1% of the 383 articles (146 articles) contained all information on the clamp procedures for which we searched.

### Participants

The 383 articles analysed included a total of 6993 participants. The median number of participants in the studies was 15 (IQR: 10–22). Most of the participants examined in the studies were healthy individuals, followed by participants with type 1 diabetes, with only a few studies enrolling people with type 2 diabetes. There was a preponderance of male participants in the studies and the participants were relatively young (Table 1).

**Table 1:** Baseline characteristics of participants

Characteristic	Type 1 diabetes	Type 2 diabetes	No diabetes
No. articles (%)	174 (45.4)	30 (7.8)	268 (70.0)
No. participants (%)	2768 (39.6)	485 (6.9)	3740 (53.5)
Age, years	31.6 ± 7.8	55.1 ± 8.4	30.7 ± 12.6
Male sex, %	62.8	67.6	65.0
HbA_1c_			
mmol/mol	65 ± 16	60 ± 13	34 ± 4
%	8.1 ± 1.4	7.7 ± 1.2	5.3 ± 0.3
Diabetes duration, years	14.3 ± 6.0	9.3 ± 5.4	
BMI, kg/m^2^	24.4 ± 1.9	29.6 ± 2.6	24.0 ± 2.2
Type 2 diabetes glucose-lowering treatment (%)			
Diet		7 (23.3)	
Oral agents alone		28 (93.3)	
Insulin		15 (50)	
Not provided		1 (3.3)	

Data are shown as *n* (%) or mean (± SD)

### Instructions and preparation

Most of the hyperinsulinaemic–hypoglycaemic clamps were scheduled in the morning. In 80.9% of all studies, participants were instructed to fast overnight prior to the clamp day. In 21.4% of the articles there were other dietary restrictions such as a weight-maintaining diet 3 days before the clamp (*n =* 40, 10.4%), a standardised meal the evening before the clamp (*n* =12, 3.1%) or a standard breakfast on the morning of the clamp (*n =* 8, 2.1%) (ESM Table 2). Participants were also instructed to abstain from drinking alcohol (13.6%), smoking tobacco (4.7%), engaging in exercise (11.5%) and ingesting caffeine (8.6%). In 23.0% of the experiments involving people with diabetes, an overnight low-dose insulin infusion was provided to normalise glucose levels prior to the clamp. In 3.9% of the experiments, participants were instructed to measure or monitor blood glucose overnight by finger stick or continuous glucose monitoring (CGM) to ensure avoidance of nocturnal hypoglycaemia, which necessitated rescheduling of the clamp.

### Clamp procedure

Human soluble insulin was used in 65.5% of the clamps, a rapid-acting analogue insulin in 1.6% and both types of insulin in 0.5%, whereas 32.4% of the articles did not report the type of insulin used (about 42.6% of which were published before market introduction of insulin analogues). Glucose (or dextrose) was administered as a 20% solution in 74.7% of the studies and as another solution in 6.5%, whereas 18.8% of the articles did not provide the glucose percentage used.

**Fig. 2:**
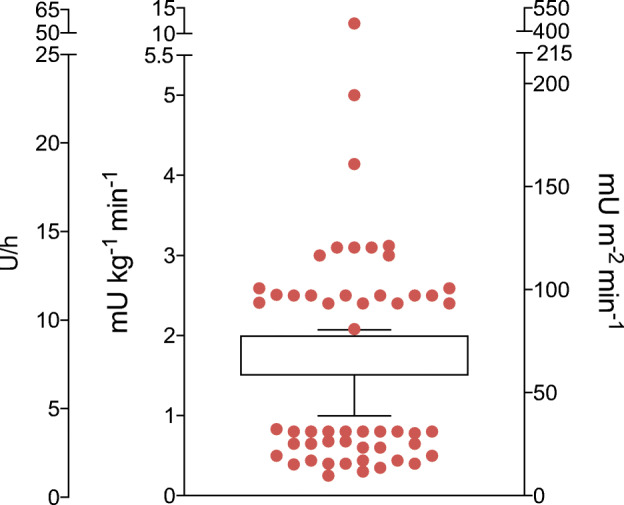
Individual IIRs are shown in a box, representing 25th–75th percentiles, and whiskers showing 10th and 90th percentiles, and are expressed in units per hour (U/h), mU kg^−1^ min^−1^ and mU m^−2^ min^−1^, normalised for a ‘standard person’ with a weight of 75 kg and a height of 180 cm

Two methods were used to calculate the IIR, i.e. based on body weight (mU or pmol kg^−1^ min^−1^) in 257 articles (67.1%) or on body surface area (BSA) (mU or pmol m^−2^ min^−1^) in 110 articles (28.7%). Sixteen articles (4.2%) did not report how the IIR was calculated. There was considerable variation in IIR across studies, ranging from 0.25 to 12.0 mU kg^−1^ min^−1^ for studies calculating IIR by body weight and from 15 to 160 mU m^−2^ min^−1^ for studies using body surface area (Fig. 2). When recalculating IIR to a person of average body weight and height (75 kg, 180 cm), mean ± SD IRR corresponded to 7.1 ± 4.1 U/h (range 1.1–54.0 U/h) based on body weight and 7.7 ± 2.6 U/h (range 1.75–18.6 U/h) based on BSA, respectively. There were no significant differences in the IIR between the single-step and stepped clamps, although in 38 (9.9%) of the latter the IIR was increased to reach the deepest hypoglycaemic level. Information about the glucose infusion rate (GIR) was provided by 24.8% of the studies.

Of the studies reporting plasma insulin levels during the clamp (*n =* 147, 38.4%), the mean CVs of these levels for IIR based on body weight and BSA were 28 ± 16 vs 32 ± 35% (*p* = 0.70), respectively.

In 89.0% of the clamps, venous blood was sampled for glucose and other measurements, whereas arterial blood was sampled in 2.3%. Four articles reported both venous and arterial blood sampling and 7.6% of the studies did not specify the source of blood sampling. Glucose was measured in plasma in 271 articles (70.8%) and in whole blood in 105 (27.4%), with 7 articles (1.8%) not providing this information. Because of the different methods of glucose measurement, we converted whole blood glucose values to plasma glucose values, assuming plasma glucose levels to be 11.1% higher compared with whole blood measurements [7]. The most widely reported location of the venous catheter for blood sampling was in a dorsal hand vein (70.9%), followed by veins in the antecubital region (6.8%), the forearm (6.2%) and the leg (0.8%). In 15.3% of the articles, the location of the catheter was not provided.

A method for arterialisation of venous blood was reported by 272 (78.8%) of the studies using venous cannulations. The methods used to arterialise venous blood varied, but the application of a heated hand box method was used most often (66.4%), followed by the use of a blanket (4.6%), a pad (1.7%), other means (1.7%) or an unspecified method (4.3%). In 21.2% of the articles, hand warming was not applied. Of all the 229 studies that used a heated box, its temperature was set at 50–60°C in 48.5%, at 60–70°C in 30.1% or at 60°C in 15.7% of the experiments. A temperature below 50°C was used in 1.3% of the articles and 4.4% did not provide the target temperature of the heated box. In two studies, arterialisation of venous blood was checked by blood gas analyses.

Glucose levels were measured at 5 min intervals in 82.5% of the studies. Shorter time intervals (down to 1.5 min) were used in 10.0% and longer intervals up to 30 min in 7.5% of the studies. Glucose levels were mostly determined with the Beckman Glucose Analyzer (39.2%) or the Yellow Spring Instruments Glucose Analyzer (36.0%). At regular intervals, blood was drawn to determine counterregulatory hormones, i.e. glucagon, catecholamines, growth hormone and cortisol, in 86.2% of the studies.

From 222 articles (58.0%), it was possible to extract (*n =*18) or calculate (*n =*204) the CV of glucose levels achieved during the clamp. In studies where this was calculated, the CV of the normoglycaemic and hypoglycaemic phases averaged 7 ± 6% and 10 ± 9%, respectively (*p* < 0.0005). The overall mean CV of the normo- and hypoglycaemic phases combined in these clamps was 8.3 ± 7%, with 72 clamps (32.4%) having a mean CV <5%, 91 (41%) a mean CV of 5–10%, 51 (23.0%) a mean CV of 11–20% and eight a mean CV of more than 20%. Of the 18 articles reporting the calculated CV, all had a CV <10%.

### Type of clamp

A single-step hypoglycaemic clamp was performed in 245 (64.0%) of the articles and a stepped clamp in 135 (35.2%), whereas three (0.8%) articles applied both single-step and stepped clamps on separate days.

#### Single-step clamp

In 192 of the 248 articles (77.4%) using single-step clamps, a normoglycaemic phase preceded the hypoglycaemic phase, the duration of which ranged from 15 to 330 min, with 30 min (23.0%), 60 min (17.7%) or 120 min (12.9%) most often used. After correction of whole blood glucose values into plasma values (see Clamp procedure), the mean plasma glucose level of the normoglycaemic phase was 5.2 ± 0.8 mmol/l. The duration of the hypoglycaemic phase ranged from 5 to 660 min; most had a duration of 30, 60 or 120 min. The mean glucose level at hypoglycaemic nadir was 2.8 ± 0.4 mmol/l, but there was considerable variation across the studies (ESM Fig. 1). In 173 articles (69.8%), the glucose nadir was <3.0 mmol/l (mean 2.7 ± 0.2 mmol/l, range 2.0–2.9), corresponding to level 2 hypoglycaemia according to the International Hypoglycaemia Study Group (IHSG) classification [8], whereas the nadir was ≥3.0 mmol/l (mean 3.2 ± 0.2 mmol/l, range 3.0–4.3) in 75 articles (30.2%).

#### Stepped clamp

Experiments using stepped clamps varied in the number of steps (range 1−6). The most frequently used number of steps after the normoglycaemic phase was four (36.2%), three (25.4%) or two (19.6%). More than four steps were used in 10.1% of articles, whereas 4.3% did not provide information on the number of steps and 4.3% had a hyperglycaemic step included.

There was a large variation in the duration of the hypoglycaemic phases for the stepped clamp studies. In the studies that used four steps, the duration ranged from 20 to 90 min per step, the majority using 45 min (33.3%), 60 min (25.0%) or 40 min (18.8%). The duration of the steps for three-step clamps ranged from 20 to 90 min per step, with 60 min (31.6%), 30 min (21.1%) or 50 min (13.2%) most frequently applied.

**Fig. 3:**
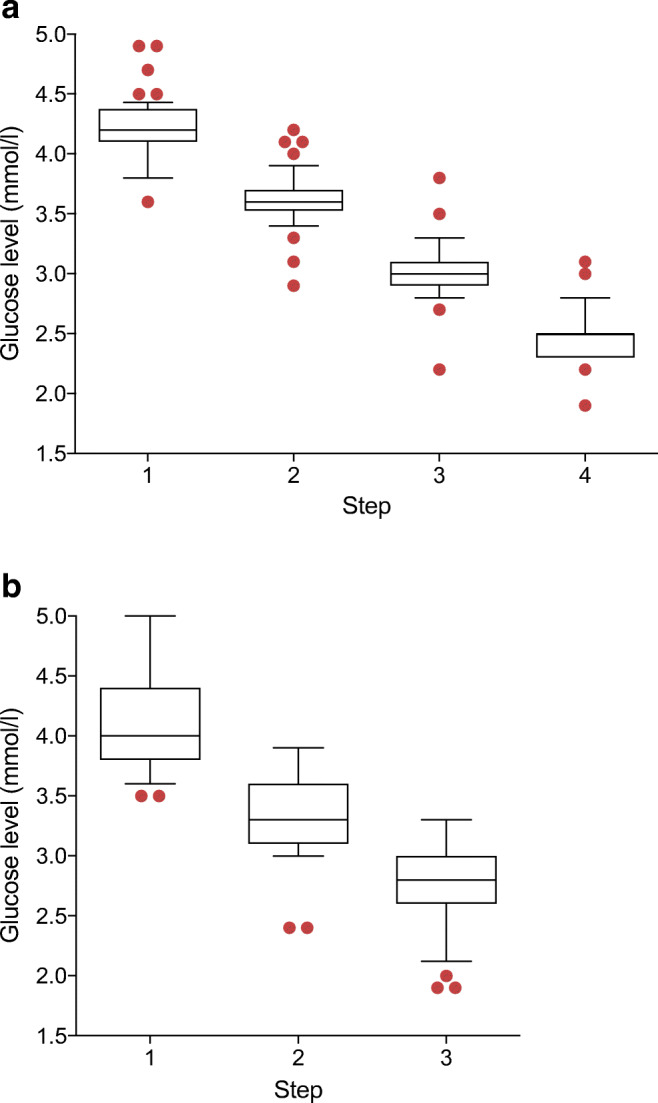
Hypoglycaemic steps in (**a**) four-stepped and (**b**) three-stepped clamps. Data are shown in a box, representing 25th–75th percentiles, and whiskers showing 10th and 90th percentiles

In the four-step clamps, the mean targeted plasma glucose levels for the consecutive steps were 4.2 ± 0.3 mmol/l, 3.6 ± 0.2 mmol/l, 3.0 ± 0.2 mmol/l and 2.5 ± 0.2 mmol/l, respectively (Fig. 3a). For the three-step clamps, these numbers averaged 4.1 ± 0.4 mmol/l, 3.4 ± 0.4 mmol/l and 2.8 ± 0.4 mmol/l (Fig. 3b). The mean glucose nadir for the stepped clamps was 2.6 ± 0.3 mmol/l (range 1.9–3.3), with 2.5 mmol/l (22.4%) or 2.8 mmol/l (16.5%) targeted most often (ESM Fig. 2). Eighty-two per cent of the studies had a glucose nadir <3.0 mmol/l (mean 2.5 ± 0.4 mmol/l, range 1.9–2.9) and 18% a nadir ≥3.0 mmol/l (mean 3.1 ± 0.1 mmol/l, range 3.0–3.3).

## Discussion

The main finding of this study is that there is substantial variation in the conduction of hyperinsulinaemic–hypoglycaemic clamps across research groups, particularly in terms of antecedent day preparation, IIRs, number of hypoglycaemic steps, hypoglycaemic nadirs and duration of the clamps. The methodology descriptions frequently lacked important information in that less than half of the articles provided all the information needed to evaluate the experiment. Clamps were usually performed in the morning, in fasting condition, with or without some form of standardisation of meals ingested the evening before the clamp. Also, most studies included instructions for people with insulin-treated diabetes to adjust insulin use to avoid (nocturnal) hypoglycaemia prior to clamping. Only one of ten studies imposed other lifestyle restrictions before the clamp, such as refraining from alcohol or caffeine intake, smoking or engaging in strenuous exercise, which can affect glucose homeostasis and responses to hypoglycaemia [9–14], although the duration of these restrictions varied from 12–24 h [15, 16] to 72 h [17, 18]. More than half of the experiments were done in participants without diabetes and only around 7% of participants had type 2 diabetes.

There was an almost 50-fold difference between the highest and lowest IIRs used during the clamps, not including the doubling of the insulin dose that some studies applied to reach the lowest glucose target in stepped clamps. In addition, many studies with participants with type 2 diabetes increased the IIR at the lowest glucose level to ensure it was maintained in the face of insulin resistance and a brisk counterregulatory response. Apart from its effect on glucose requirements, changing the ambient insulin level may affect outcomes, such as the response of counterregulatory hormones. The direction of this change is not known, with some studies observing lower counterregulatory responses of high-dose vs low-dose insulin [19] and others finding the exact opposite, albeit all in healthy men [20, 21]. Another study, performed in participants with type 1 diabetes, did not find a difference in counterregulatory hormone responses between high- or low-dose IIRs [22]. It should be noted that even the lower insulin doses are often unphysiologically elevated, as this is needed to achieve hypoglycaemia.

Whether the IIRs are calculated on the basis of body weight or BSA does not seem to be relevant. Indeed, the CVs of achieved plasma insulin levels across participants as an estimate of inter-individual insulin variability did not reveal meaningful differences between the two calculation methods. It should be noted, however, that only very few studies included obese individuals, which is relevant because obesity has a much greater effect on the calculated insulin dose when this is based on body weight rather than on BSA. Indeed, for the abovementioned person (75 kg, BSA 1.94 m^2^), the calculated insulin doses for an IIR of 60 mU m^−2^ min^−1^ or 1.5 mU kg^−1^ min^−1^ are about similar (6.98 vs 6.75 U/h), yet when this person weighs 125 kg (BSA 2.41 m^2^), these doses equal 8.68 and 11.25 U/h, respectively.

An important indicator of the quality of a glucose clamp is the CV of achieved plasma glucose levels for each glucose step. The CV reflects the stability of the glucose levels achieved during the clamp; the lower the CV, the more stable the clamp. Although there is no formal consensus on how low the CV of glucose levels should be during a hypoglycaemic clamp, a CV <5% is generally considered desirable [23]. However, this was achieved in only about a third of the articles, and it is plausible that CVs are worse in articles that neither reported nor provided the option to calculate the CV.

Soluble short-acting human insulin was used in the majority of the clamps, although some studies also used porcine insulin [24]. Since rapid-acting insulin analogues and human insulin possess the same pharmacodynamic and pharmacokinetic qualities when administered directly into the bloodstream, the choice of insulin is not considered to affect the outcome of the experiment itself. In the early days of clamp history, a priming insulin dose was often administered to quickly achieve target insulin levels. However, due to the very short *t*_½_ of insulin, such a priming dose is probably unnecessary for insulin doses below ~2 mU kg^−1^ min^−1^ (~80 mU m^−2^ min^−1^) [25] and increases the risk that glucose levels fall too quickly. For higher insulin doses, a priming dose has been calculated to shorten the time until reaching steady-state glucose disposal in normoglycaemic glucose clamps [26].

Data on GIRs were not systematically reported. Ideally, they should be reported separately for each glycaemic phase. The GIR during hypoglycaemia is a surrogate marker of the combined counterregulatory hormone response, reflecting the inverse of endogenous glucose appearance resulting from hormonal counterregulation.

Most, but not all, clamps using venous blood sampling applied some form of hand warming to achieve arterialisation of venous blood. Because insulin stimulates glucose uptake in skeletal muscle, peripheral venous samples underestimate, to a variable degree, the glucose concentrations in the blood supplying tissues, most importantly the brain. Proper hand warming opens arteriovenous shunts, resulting in arterialisation of venous blood. Liu et al. found an arteriovenous difference for high and low IIRs of 0.9 ± 0.1 and 0.4 ± 0.1 mmol/l, respectively [27], whereas the arterial–arterialised venous blood difference was about 0.1 mmol/l (95% CI −0.2, 0.4) [28]. The heated hand box method, by which the local temperature is raised in a controllable way to 55–60°C, is widely used to arterialise venous blood [28]. However, the method by which blood is arterialised is less important, as long as the temperature is sufficiently elevated. Indeed, raising the temperature to 40°C with warm blankets was found to be equally effective as the heated hand box [29]. It should be acknowledged that although the arterialisation method is reasonably well validated for glucose, this may not be the case for other compounds (e.g. counterregulatory hormones) [30], indicating that it is not possible to arterialise venous blood completely.

The vast majority of articles reported measurement of glucose levels in plasma, while the remainder of the articles reported these to be measured in whole blood. This is important, because, depending on the haematocrit, glucose levels are approximately 11% lower in whole blood than in plasma [31]. Indeed, most point-of-care glucose meters use standard algorithms to convert glucose measured in whole blood to plasma glucose. Also, the haematocrit may not be stable during clamps, which introduces bias. There is a high risk of misinterpretation when data in studies are compared without considering the source of glucose measurement from either whole blood or plasma. This is particularly relevant for the determination of hypoglycaemic thresholds, e.g. for release of counterregulatory hormones and deterioration of cognitive function, which inform decisions on the cut-offs used in the current classification for hypoglycaemia [8].

In 2017, the IHSG proposed glucose levels <3.0 mmol/l (<54 mg/dl), coined as clinically important hypoglycaemia, to be reported in clinical studies, so as to enable comparing of the effectiveness of interventions with hypoglycaemia as an endpoint [8]. The majority of the clamp studies that we investigated included a glucose level around this value, but about one of every four single-step clamps used a glucose nadir that was substantially higher (up to 4.3 mmol/l). The 3.0 mmol/l threshold level is the result of consensus and analyses are currently being conducted to refine and solidify the level [32]. For comparability reasons, it could be argued to always include such a refined threshold value in future hypoglycaemic glucose clamps, whether involving one or multiple steps.

There was also substantial variability with respect to the duration of the hypoglycaemic steps used in both the single-step and the stepped clamps. Whereas a duration of hypoglycaemia as short as 5 min (at 2.9 mmol/l) has been reported to initiate the process of habituation [33], a common protocol in clamp studies is to take approximately 20 min to reach that level and another 20 min to revert back from hypoglycaemia. The CGM definition of hypoglycaemia requires such an event to last for a minimum of 15 min, with prolonged hypoglycaemia defined as an episode of at least 120 min [34]. On the other hand, long duration of hypoglycaemia can be seen as highly unphysiological, affecting both the counterregulatory response [35] and potentially other outcomes. It seems plausible that the longer the duration of the hypoglycaemic phase, the more discomfort this may cause, so that a maximum duration of 30–60 min seems reasonable.

A total of 11 studies in this systematic review were performed in children (age range, 6.4–18.0 years), ten of which included children with type 1 diabetes. The methodology of the study protocols in the paediatric population was very similar to those of adult populations with respect to IIRs, glucose targets and overall duration. However, since the number of studies is small, extrapolating our findings in the adult population to children should be done with caution, particularly since the younger age group (<12 years) is underrepresented.

This review has limitations. Due to the large number of articles, some of which dated back >40 years, we only extracted information from the article itself and chose not to contact the authors. Also, we focused on type 1 and type 2 diabetes. Studies involving people with insulinomas [36], pancreatic transplantation [37], gastric bypass [38] or other conditions unrelated to diabetes were therefore excluded to minimise potential further methodological variability. Information about the use of albumin or the participants’ blood to prevent insulin from sticking to the infusion sets was very sparse. Similarly, very few studies provided information about the addition of potassium to the glucose/insulin infusion to avoid hypokalaemia and the potential arrhythmia-provoking consequences [39]. However, the lack of such information may suggest these adverse events to be extremely uncommon. More than 60% of the articles lacked other important information, which may reduce the validity for assessing data and comparing studies.

In conclusion, there is substantial variation in how hyperinsulinaemic–hypoglycaemic clamps have been conducted and reported in the past >40 years. This variation may potentially impact or raise questions about the validity of outcomes, and certainly makes it difficult, if not impossible, to compare results across studies. International consensus to standardise the design of both single-step and stepped clamps is therefore urgently needed.

## Supplementary information

ESM (PDF 660 kb)

## Data Availability

All data generated or analysed during this study are included in this published article (and its supplementary information files) (ESM Methods, ESM Table 1).

## Abbreviations

BSA: Body surface area
CGM: Continuous glucose monitoring
GIR: Glucose infusion rate
IHSG: International Hypoglycaemia Study Group
IIR: Insulin infusion rate
PRISMA: Preferred Reporting Items for Systematic Reviews and Meta-Analyses
