# Supplementary material for: Hyperinsulinaemic–hypoglycaemic glucose clamps in human research: a systematic review of the literature
Source: Diabetologia. 2021 Feb 10;64(4):727–36. doi: 10.1007/s00125-020-05361-8 (PMC7940281; doi:10.1007/s00125-020-05361-8)
Supplement: Supplementary file 1 — (PDF 660 kb) [file 125_2020_5361_MOESM1_ESM.pdf]

ESM Methods

Search strategy for PubMed

((((((((("hypoglycemic clamp" OR "hypoglycaemic clamp*" NOT (("hypoglycemic clamp*" OR "hypoglycaemic clamp*") AND Animals[Mesh:noexp]))) OR (("hyperinsulinemic clamp*") NOT ("hyperinsulinemic clamp*" AND Animals[Mesh:noexp]))) OR ((("glucose clamp technique"[MeSH Terms] OR "glucose clamp technique"[All Fields] OR "glucose clamp"[All Fields] OR "glucose clamps"[All Fields] OR "glucose clamping"[All Fields])) NOT (("glucose clamp technique"[MeSH Terms] OR "glucose clamp technique"[All Fields] OR "glucose clamp"[All Fields] OR "glucose clamps"[All Fields] OR "glucose clamping"[All Fields]) AND Animals[Mesh:noexp]))))))))) AND ((((((hypoglycemia OR hypoglycaemia)) NOT (((hypoglycemia) OR hypoglycaemia) AND Animals[Mesh:noexp]))) OR ((hypoglycemic OR hypoglycaemic)) NOT ((hypoglycemic OR hypoglycaemic) AND Animals[Mesh:noexp])))*

Other patient population

Studies performed in participants with the following conditions were excluded: Insulinoma (*n*=11), pituitary disease (*n*=5), pancreas transplantation (*n*=4), liver transplants (*n*=1), pancreatitis (*n*=2), patients with depression (*n*=1), severe obesity (*n*=1), fibromyalgia (*n*=1), growth hormone deficiency (*n*=2), congenital hyperinsulinaemia (*n*=1), Addison's disease (*n*=1), and adrenocortical failure (*n*=2). One article was excluded because it did not mention which participants were examined.

ESM Methods, ESM Table 1: Characteristics of the 383 articles included in this systematic review

Title	Year	Objective
Adler GK.; Bonyhay I.; Failing H.; Waring E.; Dotson S.; Freeman R.<br>Antecedent hypoglycemia impairs autonomic cardiovascular function: implications for rigorous glycemic control.<br>Diabetes Feb 2009;58(2):360-6, 2009 Feb	2009	To assess if antecedent hypoglycaemia can affect the autonomic cardiovascular function.
Adler GK.; Bonyhay I.; Curren V.; Waring E.; Freeman R.<br>Hypoglycaemia increases aldosterone in a dose-dependent fashion.<br>Diabetic medicine : a journal of the British Diabetic Association Nov 2010;27(11):1250-5, 2010 Nov	2010	To investigate the effect of hypoglycaemia on aldosterone
Abildgaard N.; Orskov L.; Petersen JA.; Alberti KG.; Schmitz O.; Møller N.<br>Forearm substrate exchange during hyperinsulinaemic hypoglycaemia in normal man.<br>Diabetic medicine : a journal of the British Diabetic Association Mar 1995;12(3):218-23, 1995 Mar	1995	To assess muscle substrate exchange during hypoglycaemia
Ahrén B.; Schweizer A.; Dejager S.; Dunning BE.; Nilsson PM.; Persson M.; Foley JE.<br>Vildagliptin enhances islet responsiveness to both hyper- and hypoglycemia in patients with type 2 diabetes.<br>The Journal of clinical endocrinology and metabolism Apr 2009;94(4):1236-43, 2009 Apr	2009	To assess the effects of the dipeptidyl peptidase-4 inhibitor vildagliptin on α-cell response to hyper- and hypoglycemia.
Allen KV.; Pickering MJ.; Zammitt NN.; Hartsuiker RJ.; Traxler MJ.; Frier BM.; Deary IJ.<br>Effects of acute hypoglycemia on working memory and language processing in adults with and without type 1 diabetes.<br>Diabetes care Jun 2015;38(6):1108-15, 2015 Jun	2015	To determine the effects of acute hypoglycemia on working memory and language processing in adults with and without type 1 diabetes.
Aman J.; Berne C.; Ewald U.; Tuvemo T.<br>Cutaneous blood flow during a hypoglycaemic clamp in insulin-dependent diabetic patients and healthy subjects.<br>Clinical science (London, England : 1979) Jun 1992;82(6):615-8, 1992 Jun	1992	To assess the effect of cutaneous blood flow in response to a gradual decline in blood glucose concentration
Amiel SA.; Pottinger RC.; Archibald HR.; Chusney G.; Cunnah DT.; Prior PF.; Gale EA.<br>Effect of antecedent glucose control on cerebral function during hypoglycemia.<br>Diabetes care Feb 1991;14(2):109-18, 1991 Feb	1991	To investigate cerebral adaption to low blood glucose level
Amiel SA.; Simonson DC.; Sherwin RS.; Lauritano AA.; Tamborlane WV.<br>Exaggerated epinephrine responses to hypoglycemia in normal and insulin-dependent diabetic children.<br>The Journal of pediatrics Jun 1987;110(6):832-7, 1987 Jun	1987	To investigate if children with insulin-dependent diabetes mellitus (IDDM) might have exaggerated hormonal responses to hypoglycemia
Amiel SA.; Simonson DC.; Tamborlane WV.; DeFronzo RA.; Sherwin RS.<br>Rate of glucose fall does not affect counterregulatory hormone responses to hypoglycemia in normal and diabetic humans.<br>Diabetes Apr 1987;36(4):518-22, 1987 Apr	1987	To assess variations in rate of glucose fall influence counterregulatory hormone responses to hypoglycemia
Amiel SA.; Maran A.; Powrie JK.; Umpleby AM.; Macdonald IA.<br>Gender differences in counterregulation to hypoglycaemia.<br>Diabetologia May 1993;36(5):460-4, 1993 May	1993	To assess the effect of gender on catecholamine

		responses to hypoglycaemia
Amiel SA.; Sherwin RS.; Simonson DC.; Tamborlane WV.<br>Effect of intensive insulin therapy on glycemic thresholds for counterregulatory hormone release.<br>Diabetes Jul 1988;37(7):901-7, 1988 Jul	1988	To investigate the influence of improved glycemic control of diabetes on plasma glucose thresholds for initiating counterregulatory hormone release.
Ang M.; Meyer C.; Brendel MD.; Bretzel RG.; Linn T.<br>Magnitude and mechanisms of glucose counterregulation following islet transplantation in patients with type 1 diabetes suffering from severe hypoglycaemic episodes.<br>Diabetologia Mar 2014;57(3):623-32, 2014 Mar	2014	To assess the effect of islet transplantation with partial graft function on glucose level.
Banarer S.; McGregor VP.; Cryer PE.<br>Intraislet hyperinsulinemia prevents the glucagon response to hypoglycemia despite an intact autonomic response.<br>Diabetes Apr 2002;51(4):958-65<br>2002 Apr	2002	To determine if intraislet hyperinsulinemia prevents the glucagon response to hypoglycemia despite an intact autonomic response
Bahlmann L.; Oltmanns KM.; Peters A.; Poeling J.; Schwemmer U.; Heringlake M.; Klaus S.<br>Metabolic stress during hypoglycaemia clamp assessed by microdialysis.<br>Minerva anestesiologica Nov 2005;71(11):711-6<br>2005 Nov	2005	To investigate subcutaneous and blood microdialysis during hypoglycaemic clamp
Bao S.; Briscoe VJ.; Tate DB.; Davis SN.<br>Effects of differing antecedent increases of plasma cortisol on counterregulatory responses during subsequent exercise in type 1 diabetes.<br>Diabetes Sep 2009;58(9):2100-8<br>2009 Sep	2009	To study whether antecedent increase of plasma cortisol is a mechanism for blunting of neuroendocrine and autonomic nervous system responses to next-day exercise in type 1 diabetes
Barrou Z.; Seaquist ER.; Robertson RP.<br>Pancreas transplantation in diabetic humans normalizes hepatic glucose production during hypoglycemia.<br>Diabetes May 1994;43(5):661-6<br>1994 May	1994	To study the effects of pancreas transplantation on counterregulation of hypoglycemia is of vital clinical importance
Battezzati A.; Benedini S.; Sereni LP.; DeTaddeo F.; Maffi P.; Secchi A.; Luzi L.<br>Protein and glutamine kinetics during counter-regulatory failure in type 1 diabetes.<br>Nutrition, metabolism, and cardiovascular diseases : NMCD Jun 2009;19(5):352-7, 2009 Jun	2009	To assess whether glutamine and amino acid utilization during hypoglycaemia is altered in T1DM with defective counter-regulatory responses.
Battezzati A.; Benedini S.; Fattorini A.; Piceni Sereni L.; Luzi L.<br>Effect of hypoglycemia on amino acid and protein metabolism in healthy humans.<br>Diabetes Sep 2000;49(9):1543-51, 2000 Sep	2000	To investigate how hypoglycaemia affects glucose and lipid metabolism
Belfort-DeAguiar RD.; Naik S.; Hwang J.; Szepietowska B.; Sherwin RS.<br>Inhaled Formoterol Diminishes Insulin-Induced Hypoglycemia in Type 1 Diabetes.<br>Diabetes care Sep 2015;38(9):1736-41, 2015 Sep	2015	To determine if long-acting inhaled β-2 AR agonists could potentially be used to treat or prevent hypoglycemia has not been established.

Belfort-DeAguiar R.; Gallezot JD.; Hwang JJ.; Elshafie A.; Yeckel CW.; Chan O.; Carson RE.; Ding YS.; Sherwin RS.<br>Noradrenergic Activity in the Human Brain: A Mechanism Supporting the Defense Against Hypoglycemia.<br>The Journal of clinical endocrinology and metabolism Jun 2018;103(6):2244-2252 2018 Jun	2018	To Investigate the potential role of brain noradrenergic activation in humans during hypoglycemia.
Bingham E.; Hopkins D.; Pernet A.; Reid H.; Macdonald IA.; Amiel SA.<br>The effects of KATP channel modulators on counterregulatory responses and cognitive function during acute controlled hypoglycaemia in healthy men: a pilot study.<br>Diabetic medicine : a journal of the British Diabetic Association Mar 2003;20(3):231-7 2003 Mar	2003	To study the effects of agents that alter potassium adenosine triphosphate (KATP) channel activity in β-cells on cognitive function and counterregulatory hormone responses during acute hypoglycaemia
Bischof MG.; Mlynarik V.; Brehm A.; Bernroider E.; Krssak M.; Bauer E.; Madl C.; Bayerle-Eder M.; Waldhäusl W.; Roden M.<br>Brain energy metabolism during hypoglycaemia in healthy and type 1 diabetic subjects.<br>Diabetologia Apr 2004;47(4):648-51, 2004 Apr	2004	To examine brain energy metabolism during moderate insulin-induced hypoglycaemia
Bischof MG.; Ludwig C.; Hofer A.; Kletter K.; Krebs M.; Stingl H.; Nowotny P.; Waldhäusl W.; Roden M.<br>Hormonal and metabolic counterregulation during and after high-dose insulin-induced hypoglycemia in diabetes mellitus type 2.<br>Hormone and metabolic research = Hormon- und Stoffwechselforschung = Hormones et metabolisme Oct 2000;32(10):417-23, 2000 Oct	2000	To compare hypoglycemia counterregulation of healthy and type 2 diabetic subjects in good metabolic control and to examine the secretion of IAPP and leptin during and following hypoglycemia in healthy and type 2 diabetic subjects.
Björklund AO.; Adamson UK.; Almström NH.; Enocksson EA.; Gennser GM.; Lins PE.; Westgren LM.<br>Effects of hypoglycaemia on fetal heart activity and umbilical artery Doppler velocity waveforms in pregnant women with insulin-dependent diabetes mellitus.<br>British journal of obstetrics and gynaecology May 1996;103(5):413-20, 1996 May	1996	To study the effect of hypoglycaemia on fetal wellbeing as indicated by fetal heart rate and umbilical flow velocity
Blackman JD.; Towle VL.; Lewis GF.; Spire JP.; Polonsky KS.<br>Hypoglycemic thresholds for cognitive dysfunction in humans.<br>Diabetes Jul 1990;39(7):828-35, 1990 Jul	1990	To investigate if hypoglycemia induces abnormalities in decision-making processes
Blauw H.; Wendl I.; DeVries JH.; Heise T.; Jax T.; .<br>Pharmacokinetics and pharmacodynamics of various glucagon dosages at different blood glucose levels.<br>Diabetes, obesity & metabolism Jan 2016;18(1):34-9, 2016 Jan	2016	To study the pharmacokinetics and pharmacodynamics of different doses of glucagon administered subcutaneously (s.c.) at different blood glucose levels
Bokhari S.; Plummer E.; Emmerson P.; Gupta A.; Meyer C.<br>Glucose counterregulation in advanced type 2 diabetes: effect of β-adrenergic blockade.<br>Diabetes care Nov 2014;37(11):3040-6, 2014 Nov	2014	To examine counterregulatory glucose kinetics in advanced DM2 and test the hypothesis that these are impaired by β-adrenergic blockade.

Bolo NR.; Musen G.; Simonson DC.; Nickerson LD.; Flores VL.; Siracusa T.; Hager B.; Lyoo IK.; Renshaw PF.; Jacobson AM.<br>Functional Connectivity of Insula, Basal Ganglia, and Prefrontal Executive Control Networks during Hypoglycemia in Type 1 Diabetes.<br>The Journal of neuroscience : the official journal of the Society for Neuroscience Aug 2015;35(31):11012-23, 2015 Aug	2015	To Investigate glucose effects independently og insulin effects in the brain
Bolli G.; De Feo P.; Perriello G.; De Cosmo S.; Ventura M.; Campbell P.; Brunetti P.; Gerich JE.<br>Role of hepatic autoregulation in defense against hypoglycemia in humans.<br>The Journal of clinical investigation May 1985;75(5):1623-31, 1985 May	1985	To assess the role of hepatic autoregulation in defense against hypoglycemia,
Boyle PJ.; Schwartz NS.; Shah SD.; Clutter WE.; Cryer PE.<br>Plasma glucose concentrations at the onset of hypoglycemic symptoms in patients with poorly controlled diabetes and in nondiabetics.<br>The New England journal of medicine Jun 1988;318(23):1487-92<br>1988 Jun	1988	To study if symptoms of hypoglycemia occur at higher glucoseconcentr ations in patients with p oorly controlled insulin-dependent diabetes me llitus than in persons without diabetes
Boyle PJ.; Nagy RJ.; O'Connor AM.; Kempers SF.; Yeo RA.; Qualls C.<br>Adaptation in brain glucose uptake following recurrent hypoglycemia.<br>Proceedings of the National Academy of Sciences of the United States of America Sep 1994;91(20):9352-6,1994 Sep	1994	To investigate if adaptations occur to allow maintenance of normal rates of brain glucose uptake following recurrent hypoglycemia in man
Brierley EJ.; Broughton DL.; James OF.; Alberti KG.<br>Reduced awareness of hypoglycaemia in the elderly despite an intact counter-regulatory response.<br>QJM : monthly journal of the Association of Physicians Jun 1995;88(6):439-45, 1995 Jun	1995	To study awareness of hypoglycaemia and its counterregulatory hormones
Briscoe VJ.; Ertl AC.; Tate DB.; Davis SN.<br>Effects of the selective serotonin reuptake inhibitor fluoxetine on counterregulatory responses to hypoglycemia in individuals with type 1 diabetes.<br>Diabetes Dec 2008;57(12):3315-22, 2008 Dec	2008	To test the hypothesis that 6-week use of the SSRI fluoxetine would amplify autonomic nervous system (ANS) counterregulatory responses to hypoglycemia in individuals with type 1 diabetes.
Briscoe VJ.; Ertl AC.; Tate DB.; Dawling S.; Davis SN.<br>Effects of a selective serotonin reuptake inhibitor, fluoxetine, on counterregulatory responses to hypoglycemia in healthy individuals.<br>Diabetes Sep 2008;57(9):2453-60, 2008 Sep	2008	To test the hypothesis that 6 weeks' administration of the selective serotonin reuptake inhibitor (SSRI) fluoxetine would amplify autonomic nervous system (ANS) and neuroendocrine counter-regulatory mechanisms during hypoglycemia.
Bremer JP.; Baron M.; Peters H.; Oltmanns KM.; Kern W.; Fehm HL.; Born J.; Schultes B.<br>Hormonal, subjective, and neurocognitive responses to brief hypoglycemia in postmenopausal women and age-matched men with type 2 diabetes mellitus.<br>Metabolism: clinical and experimental Mar 2006;55(3):331-8, 2006 Mar	2006	To determine aex differences in counterregulation are present also in type 2 diabetic patients who are in postmenopausal state
Brody S.; Keller U.; Degen L.; Cox DJ.; Schächinger H.	2004	To assess whether selective cognitive

Selective processing of food words during insulin-induced hypoglycemia in healthy humans.<br>Psychopharmacology Apr 2004;173(1-2):217-20<br>2004 Apr		processing of food stimuli occurs during insulin-induced hypoglycemia
Caduff A.; Lutz HU.; Heinemann L.; Di Benedetto G.; Talary MS.; Theander S.<br>Dynamics of blood electrolytes in repeated hyper- and/or hypoglycaemic events in patients with type 1 diabetes.<br>Diabetologia Oct 2011;54(10):2678-89 2011 Oct	2011	To study ionic profiles of patients with type 1 diabetes during consecutive hyper- and/or hypoglycaemic events using the glucose clamp
Cade WT.; Khoury N.; Nelson S.; Shackleford A.; Semenkovich K.; Krauss MJ.; Arbeláez AM.<br>Hypoglycemia during moderate intensity exercise reduces counterregulatory responses to subsequent hypoglycemia.<br>Physiological reports 09 2016;4(17): 2016 09	2016	To study dextrose administration to maintain euglycemia during moderate intensity exercise alters the attenuation of counterregulatory responses to subsequent hypoglycemia in healthy adults
Caprio S.; Amiel S.; Tamborlane WV.; Gelfand RA.; Sherwin RS.<br>Defective free-fatty acid and oxidative glucose metabolism in IDDM during hypoglycemia. Influence of glycemic control.<br>Diabetes Feb 1990;39(2):134-41, 1990 Feb'	1990	To assess the impact of diabetes and its treatment on plasma free-fatty acid and oxidative fuel metabolism during hypoglycemia
Caprio S.; Tamborlane WV.; Zych K.; Gerow K.; Sherwin RS.<br>Loss of potentiating effect of hypoglycemia on the glucagon response to hyperaminoacidemia in IDDM.<br>Diabetes Apr 1993;42(4):550-5, 1993 Apr	1993	To test whether raising amino acid levels during hypoglycemia could reverse the defective a-cell response in IDDM patients
Carey M.; Gospin R.; Goyal A.; Tomuta N.; Sandu O.; Mbanya A.; Lontchi-Yimagou E.; Hulkower R.; Shamoon H.; Gabriely I.; Hawkins M.<br>Opioid Receptor Activation Impairs Hypoglycemic Counterregulation in Humans.<br>Diabetes 11 2017;66(11):2764-2773, 2017 11	2017	To determine whether and how opioid receptor activation induces HAAF in humans
Cersosimo E.; Garlick P.; Ferretti J.<br>Renal glucose production during insulin-induced hypoglycemia in humans.<br>Diabetes Feb 1999;48(2):261-6, 1999 Feb	1999	To assess the effects of hypoglycemia on renal glucose production and renal glucose uptake using arteriovenous balance combined with tracer technique in humans.
Chalew SA.; Sakamoto RN.; McCarter R.; Hanukoglu A.; Kowarski AA.; Matjasko J.<br>Quantitative monitoring of brain function, vital signs, and hormonal response during acute insulin-induced hypoglycemia.<br>Journal of clinical monitoring Oct 1989;5(4):229-35<br>1989 Oct	1989	To study quantitative monitoring of brain function, vital signs, and hormonal response during acute insulin-induced hypoglycemia
Chan A.; Heinemann L.; Anderson SM.; Breton MD.; Kovatchev BP.<br>Nonlinear metabolic effect of insulin across the blood glucose range in patients with type 1 diabetes mellitus.<br>Journal of diabetes science and technology Jul 2010;4(4):873-81<br>2010 Jul	2010	To assess the ability of insulin to lower BG in patients with DM1 in hypoglycemia and hyperglycemia.

Cheyne EH.; Sherwin RS.; Lunt MJ.; Cavan DA.; Thomas PW.; Kerr D.<br>Influence of alcohol on cognitive performance during mild hypoglycaemia; implications for Type 1 diabetes.<br>Diabetic medicine : a journal of the British Diabetic Association Mar 2004;21(3):230-7, 2004 Mar	2004	To examine the effect of mild hypoglycaemia (2.8 mmol/l) with modest alcohol intoxication (levels below driving limits) on intellectual performance in patients with Type 1 diabetes
Cheyne EH.; Cavan DA.; Kerr D.<br>Performance of a continuous glucose monitoring system during controlled hypoglycaemia in healthy volunteers.<br>Diabetes technology & therapeutics 2002;4(5):607-13, 2002	2002	To study CGM with YSI during hypo clamp
Chow E.; Bernjak A.; Walkinshaw E.; Lubina-Solomon A.; Freeman J.; Macdonald IA.; Sheridan PJ.; Heller SR.<br>Cardiac Autonomic Regulation and Repolarization During Acute Experimental Hypoglycemia in Type 2 Diabetes.<br>Diabetes 05 2017;66(5):1322-1333, 2017 05	2017	To study changes in cardiac autonomic function and repolarization during sustained experimental hypoglycemia
Chow E.; Iqbal A.; Walkinshaw E.; Phoenix F.; Macdonald IA.; Storey RF.; Ajjan R.; Heller SR.<br>Prolonged Prothrombotic Effects of Antecedent Hypoglycemia in Individuals With Type 2 Diabetes.<br>Diabetes care Oct 2018;(): 2018 Oct	2018	To examine acute and downstream effects of hypoglycemia on markers of thrombosis risk and inflammation in type 2 diabetes
Choudhary P.; Lonnen K.; Emery CJ.; MacDonald IA.; MacLeod KM.; Amiel SA.; Heller SR.<br>Comparing hormonal and symptomatic responses to experimental hypoglycaemia in insulin- and sulphonylurea-treated Type 2 diabetes.<br>Diabetic medicine : a journal of the British Diabetic Association Jul 2009;26(7):665-72, 2009 Jul	2009	To study physiological response to hypoglycaemia in DM2 patients treated with sulphonylureas or insulin compared with HP
Christensen MB.; Calanna S.; Holst JJ.; Vilsbøll T.; Knop FK.<br>Glucose-dependent insulinotropic polypeptide: blood glucose stabilizing effects in patients with type 2 diabetes.<br>The Journal of clinical endocrinology and metabolism Mar 2014;99(3):E418-26, 2014 Mar	2014	To evaluate the importance of the prevailing plasma glucose levels for the effect of GIP on responses of glucagon and insulin and glucose disposal in patients with DM2
Clowes JA.; Robinson RT.; Heller SR.; Eastell R.; Blumsohn A.<br>Acute changes of bone turnover and PTH induced by insulin and glucose: euglycemic and hypoglycemic hyperinsulinemic clamp studies.<br>The Journal of clinical endocrinology and metabolism Jul 2002;87(7):3324-9, 2002 Jul	2002	To study the effect of euglycemic hyperinsulinemia and hypoglycemic hyperinsulinemia on bone turnover using an insulin clamp
Cohen O.; Fine I.; Monashkin E.; Karasik A.<br>Glucose correlation with light scattering patterns--a novel method for non-invasive glucose measurements.<br>Diabetes technology & therapeutics 2003;5(1):11-7, 2003	2003	To correlate occlusion red near-infrared spectroscopy technology with intravenous and interstitial glucose levels, occlusion red near-infrared spectroscopy and glucose levels
Cooperberg BA.; Cryer PE.<br>Insulin reciprocally regulates glucagon secretion in humans.<br>Diabetes Nov 2010;59(11):2936-40, 2010 Nov	2010	To study if increase in insulin per se, i.e., in the absence of zinc,

		suppresses glucagon secretion during euglycemia and that a decrease in insulin per se stimulates glucagon secretion during hypoglycemia in humans
Cox DJ.; Gonder-Frederick LA.; Kovatchev BP.; Clarke WL.<br>Self-treatment of hypoglycemia while driving.<br>Diabetes research and clinical practice Oct 2001;54(1):17-26, 2001 Oct	2001	To assess self-treatment of hypoglycaemia while driving
Cranston I.; Lomas J.; Maran A.; Macdonald I.; Amiel SA.<br>Restoration of hypoglycaemia awareness in patients with long-duration insulin-dependent diabetes.<br>Lancet (London, England) Jul 1994;344(8918):283-7, 1994 Jul	1994	To investigate the possibility of restoring awareness; symptomatic, cognitive, and hormonal responses to controlled hypoglycaemia were studied in insulin-dependent diabetic patients with long disease duration
Cranston I.; Reed LJ.; Marsden PK.; Amiel SA.<br>Changes in regional brain (18)F-fluorodeoxyglucose uptake at hypoglycemia in type 1 diabetic men associated with hypoglycemia unawareness and counter-regulatory failure.<br>Diabetes Oct 2001;50(10):2329-36, 2001 Oct	2001	To examine changes in regional bran (18) F-fluorodeoxyglucose during hypoglycaemia for unaware DM1´s
Criego AB.; Tkac I.; Kumar A.; Thomas W.; Gruetter R.; Seaquist ER.<br>Brain glucose concentrations in healthy humans subjected to recurrent hypoglycemia.<br>Journal of neuroscience research Nov 2005;82(4):525-30 2005 Nov.	2005	To study whether the brain glucose concentration is altered in normal subjects subjected to recurrent hypoglycemia
Dantz D.; Bewersdorf J.; Fruehwald-Schultes B.; Kern W.; Jelkmann W.; Born J.; Fehm HL.; Peters A.<br>Vascular endothelial growth factor: a novel endocrine defensive response to hypoglycemia.<br>The Journal of clinical endocrinology and metabolism Feb 2002;87(2):835-40, 2002 Feb	2002	To investigate if vascular endothelial growth factor (VEGF), is a potent regulator of blood vessel function, is a candidate hormone for facilitating glucose passage across the blood-brain barrier under critical conditions
Dagogo-Jack SE.; Craft S.; Cryer PE.<br>Hypoglycemia-associated autonomic failure in insulin-dependent diabetes mellitus. Recent antecedent hypoglycemia reduces autonomic responses to, symptoms of, and defense against subsequent hypoglycemia.<br>The Journal of clinical investigation Mar 1993;91(3):819-28, 1993 Mar	1993	To study if patients with insulin-dependent diabetes mellitus (IDDM), recent antecedent iatrogenic hypoglycemia is a major cause of hypoglycemia-associated autonomic failure
Dagogo-Jack S.; Askari H.; Morrill B.; Lehner LL.; Kim B.; Sha X.<br>Physiological responses during hypoglycaemia induced by regular human insulin or a novel human analogue, insulin glargine.<br>Diabetes, obesity & metabolism Dec 2000;2(6):373-83, 2000 Dec	2000	To determine and compare insulin glargine and regular insulin in relation to hypoglycaemic symptoms
Dagogo-Jack S.; Rattarasarn C.; Cryer PE.	1994	To study neuroendocrine

Reversal of hypoglycemia unawareness, but not defective glucose counterregulation, in IDDM.<br>Diabetes Dec 1994;43(12):1426-34, 1994 Dec		(including autonomic) responses to hypoglycemia are dissociated from the symptomatic responses to hypoglycemia in insulin-dependent diabetes mellitus
Davis MR.; Mellman M.; Shamoon H.<br>Further defects in counterregulatory responses induced by recurrent hypoglycemia in IDDM.<br>Diabetes Oct 1992;41(10):1335-40, 1992 Oct	1992	To assess whether the defects that we previously reported to be inducible by repeated hypoglycemia in nondiabetic subjects could also occur in people with IDDM
Davis MR.; Shamoon H.<br>Impaired glucose disposal following mild hypoglycemia in nondiabetic and type I diabetic humans.<br>Metabolism: clinical and experimental Feb 1992;41(2):216-23, 1992 Feb	1992	To study insulin-mediated glucose disposal was studied immediately prior to and following moderate hypoglycemia
Davis MR.; Mellman M.; Shamoon H.<br>Physiologic hyperinsulinemia enhances counterregulatory hormone responses to hypoglycemia in IDDM.<br>The Journal of clinical endocrinology and metabolism May 1993;76(5):1383-5, 1993 May	1993	To evaluate the effect of physiologic hyperinsulinemia on counterregulatory hormone responses in 8 IDDM subjects
Davis SN.; Shavers C.; Costa F.<br>Differential gender responses to hypoglycemia are due to alterations in CNS drive and not glycemic thresholds.<br>American journal of physiology. Endocrinology and metabolism Nov 2000;279(5):E1054-63, 2000 Nov	2000	To determine whether differential glycemic thresholds are the mechanism responsible for the sexual dimorphism present in counterregulatory responses during hypoglycemia, and to determine the integrated physiological responses that occur over a range of mild to moderate hypoglycemia in healthy men and women.
Davis SN.; Cherrington AD.; Goldstein RE.; Jacobs J.; Price L.<br>Effects of insulin on the counterregulatory response to equivalent hypoglycemia in normal females.<br>The American journal of physiology Nov 1993;265(5 Pt 1):E680-9<br>1993 Nov	1993	To determine if insulin could augment the counterregulatory response to equivalent hypoglycemia in normal females similarly to males
Davis SN.; Shavers C.; Collins L.; Cherrington AD.; Price L.; Hedstrom C.<br>Effects of physiological hyperinsulinemia on counterregulatory response to prolonged hypoglycemia in normal humans.<br>The American journal of physiology Sep 1994;267(3 Pt 1):E402-10<br>1994 Sep	1994	To study if differing physiological insulin levels can modify the counterregulatory response to prolonged hypoglycemia,
Davis SN.; Shavers C.; Costa F.; Mosqueda-Garcia R.<br>Role of cortisol in the pathogenesis of deficient counterregulation after antecedent hypoglycemia in normal humans.<br>The Journal of clinical investigation Aug 1996;98(3):680-91	1996	To determine whether hypoglycemia associated autonomic failure is caused by antecedent increases in

1996 Aug		plasma cortisol in normal humans
Davis SN.; Fowler S.; Costa F.<br>Hypoglycemic counterregulatory responses differ between men and women with type 1 diabetes.<br>Diabetes Jan 2000;49(1):65-72, 2000 Jan	2000	To determine whether sex-related differences occur in counterregulatory responses to hypoglycemia in adult type 1 diabetic patients
Davis SN.; Goldstein RE.; Jacobs J.; Price L.; Wolfe R.; Cherrington AD.<br>The effects of differing insulin levels on the hormonal and metabolic response to equivalent hypoglycemia in normal humans.<br>Diabetes Feb 1993;42(2):263-72, 1993 Feb	1993	To determine whether the duration of antecedent hypoglycemia regulates the magnitude of subsequent counterregulatory failure
Davis SN.; Mann S.; Briscoe VJ.; Ertl AC.; Tate DB.<br>Effects of intensive therapy and antecedent hypoglycemia on counterregulatory responses to hypoglycemia in type 2 diabetes.<br>Diabetes Mar 2009;58(3):701-9, 2009 Mar	2009	To test the hypothesis that 1) 6 months of intensive therapy to lower A1C <7.0% would blunt autonomic nervous system responses to hypoglycemia, and 2) antecedent hypoglycemia will result in counterregulatory failure during subsequent hypoglycemia in patients with suboptimal and good glycemic control.
Davis SN.; Goldstein RE.; Price L.; Jacobs J.; Cherrington AD.<br>The effects of insulin on the counterregulatory response to equivalent hypoglycemia in patients with insulin-dependent diabetes mellitus.<br>The Journal of clinical endocrinology and metabolism Nov 1993;77(5):1300-7, 1993 Nov	1993	To determine if differing concentrations of insulin can modify the counterregulatory response to equivalent fixed hypo in insulin-dependent diabetic patients
Davis SN.; Mann S.; Galassetti P.; Neill RA.; Tate D.; Ertl AC.; Costa F.<br>Effects of differing durations of antecedent hypoglycemia on counterregulatory responses to subsequent hypoglycemia in normal humans.<br>Diabetes Nov 2000;49(11):1897-903, 2000 Nov	2000	To determine whether the duration of antecedent hypoglycemia regulates the magnitude of subsequent counterregulatory failure
Davis MR.; Shamoon H.<br>Counterregulatory adaptation to recurrent hypoglycemia in normal humans.<br>The Journal of clinical endocrinology and metabolism Nov 1991;73(5):995-1001, 1991 Nov	1991	To study the effect of antecedent hypoglycemia on glucose counterregulation during hypoglycemia in nondiabetic human subject
Debrah K.; Sherwin RS.; Murphy J.; Kerr D.<br>Effect of caffeine on recognition of and physiological responses to hypoglycaemia in insulin-dependent diabetes.<br>Lancet (London, England) Jan 1996;347(8993):19-24, 1996 Jan	1996	To investigate the effects of caffeine ingestion (250 mg) on the brain

De Feo P.; Perriello G.; De Cosmo S.; Ventura MM.; Campbell PJ.; Brunetti P.; Gerich JE.; Bolli GB.<br>Comparison of glucose counterregulation during short-term and prolonged hypoglycemia in normal humans.<br>Diabetes May 1986;35(5):563-9, 1986 May	1986	To compare glucose counterregulatory mechanisms during short-term hypoglycemia and prolonged hypoglycemia
de Galan BE.; Tack CJ.; Lenders JW.; Pasman JW.; Elving LD.; Russel FG.; Lutterman JA.; Smits P.<br>Theophylline improves hypoglycemia unawareness in type 1 diabetes.<br>Diabetes Mar 2002;51(3):790-6, 2002 Mar	2002	To test the effect of theophylline on responses to hypoglycemia
de Galan BE.; Netea MG.; Smits P.; van der Meer JW.<br>Hypoglycaemia downregulates endotoxin-induced production of tumour necrosis factor-alpha, but does not affect IL-1beta, IL-6, or IL-10.<br>Cytokine May 2003;22(3-4):71-6, 2003 May		To investigate the effect of hypoglycaemia on the production of the proinflammatory cytokines tumour necrosis factor-a (TNFa) and interleukin-1b (IL-1b) in subjects with and without diabetes
de Galan BE.; Tack CJ.; Lenders JW.; Lutterman JA.; Smits P.<br>Effect of 2 weeks of theophylline on glucose counterregulation in patients with type 1 diabetes and unawareness of hypoglycemia.<br>Clinical pharmacology and therapeutics Jul 2003;74(1):77-84<br>2003 Jul	2003	To assess the effect of theophylline oral 3 weeks prior to clamp
Degn KB.; Brock B.; Juhl CB.; Djurhuus CB.; Grubert J.; Kim D.; Han J.; Taylor K.; Fineman M.; Schmitz O.<br>Effect of intravenous infusion of exenatide (synthetic exendin-4) on glucose-dependent insulin secretion and counterregulation during hypoglycemia.<br>Diabetes Sep 2004;53(9):2397-403, 2004 Sep	2004	To assess the effects of a continuous intravenous infusion of exenatide on the counterregulatory response to hypoglycemia
De Feyter HM.; Mason GF.; Shulman GI.; Rothman DL.; Petersen KF.<br>Increased brain lactate concentrations without increased lactate oxidation during hypoglycemia in type 1 diabetic individuals.<br>Diabetes Sep 2013;62(9):3075-80, 2013 Sep	2013	To examine the transport of [3-13C]lactate across the blood-brain barrier and its metabolism in the brain
Deininger E.; Oltmanns KM.; Wellhoener P.; Fruehwald-Schultes B.; Kern W.; Heuer B.; Dominiak P.; Born J.; Fehm HL.; Peters A.<br>Losartan attenuates symptomatic and hormonal responses to hypoglycemia in humans.<br>Clinical pharmacology and therapeutics Oct 2001;70(4):362-9, 2001 Oct	2001	Investigate the effects of losartan on symptomatic and hormonal responses to hypoglycaemis in humans
Diamond MP.; Hallarman L.; Starick-Zych K.; Jones TW.; Connolly-Howard M.; Tamborlane WV.; Sherwin RS.<br>Suppression of counterregulatory hormone response to hypoglycemia by insulin per se.<br>The Journal of clinical endocrinology and metabolism Jun 1991;72(6):1388-90, 1991 Jun	1991	To study the exogenous insulin dose on the hormone response
Diamond MP.; Jones T.; Caprio S.; Hallarman L.; Diamond MC.; Addabbo M.; Tamborlane WV.; Sherwin RS.<br>Gender influences counterregulatory hormone responses to hypoglycemia.<br>Metabolism: clinical and experimental Dec 1993;42(12):1568-72<br>1993 Dec	1993	To asses if counterregulatory hormone responses to hypoglycemia is influenced by gender
Diamond MP.; Grainger DA.; Rossi G.; Connolly-Diamond M.; Sherwin RS.<br>Counter-regulatory response to hypoglycemia in the follicular and luteal phases of the menstrual cycle.<br>Fertility and sterility Dec 1993;60(6):988-93, 1993 Dec	1993	To assess whether the phase of the menstrual cycle influences the counter-regulatory response to hypoglycemia

Dotson S.; Freeman R.; Failing HJ.; Adler GK.<br>Hypoglycemia increases serum interleukin-6 levels in healthy men and women.<br>Diabetes care Jun 2008;31(6):1222-3, 2008 Jun	2008	To investigate the effect of hypoglycemia on IL-6
Enoksson S.; Caprio SK.; Rife F.; Shulman GI.; Tamborlane WV.; Sherwin RS.<br>Defective activation of skeletal muscle and adipose tissue lipolysis in type 1 diabetes mellitus during hypoglycemia.<br>The Journal of clinical endocrinology and metabolism Apr 2003;88(4):1503-11, 2003 Apr	2003	The effect of microdialysis to monitor glycerol (index of lipolysis) in the extracellular fluid
Ertl AC.; Mann S.; Richardson A.; Briscoe VJ.; Blair HB.; Tate DB.; Davis SN.<br>Effects of oral carbohydrate on autonomic nervous system counterregulatory responses during hyperinsulinemic hypoglycemia and euglycemia.<br>American journal of physiology. Endocrinology and metabolism Sep 2008;295(3):E618-25, 2008 Sep	2008	To determine the effects of oral carbohydrate on autonomic nervous system (ANS) and neuroendocrine responses
Evans ML.; Matyka K.; Lomas J.; Pernet A.; Cranston IC.; Macdonald I.; Amiel SA.<br>Reduced counterregulation during hypoglycemia with raised circulating nonglucose lipid substrates: evidence for regional differences in metabolic capacity in the human brain?<br>The Journal of clinical endocrinology and metabolism Aug 1998;83(8):2952-9, 1998 Aug	1998	To assess the potential for the human brain to use lipid fuels during acute hypoglycemia
Evans ML.; Pernet A.; Lomas J.; Jones J.; Amiel SA.<br>Delay in onset of awareness of acute hypoglycemia and of restoration of cognitive performance during recovery.<br>Diabetes care Jul 2000;23(7):893-7, 2000 Jul	2000	To examine the time course for the onset of, and recovery from, acute hypoglycemia.
Evans ML.; Hopkins D.; Macdonald IA.; Amiel SA.<br>Alanine infusion during hypoglycaemia partly supports cognitive performance in healthy human subjects.<br>Diabetic medicine : a journal of the British Diabetic Association May 2004;21(5):440-6, 2004 May	2004	To determine the potential for the non-glucose metabolic substrate alanine to support brain function during glucose deprivation
Ewing FM.; Deary IJ.; McCrimmon RJ.; Strachan MW.; Frier BM.<br>Effect of acute hypoglycemia on visual information processing in adults with type 1 diabetes mellitus.<br>Physiology & behavior Jul 1998;64(5):653-60, 1998 Jul	1998	The effect of acute hypoglycaemia on visual information processing
Fanelli C.; Pampanelli S.; Lalli C.; Del Sindaco P.; Ciofetta M.; Lepore M.; Porcellati F.; Bottini P.; Di Vincenzo A.; Brunetti P.; Bolli GB.<br>Long-term intensive therapy of IDDM patients with clinically overt autonomic neuropathy: effects on hypoglycemia awareness and counterregulation.<br>Diabetes Jul 1997;46(7):1172-81, 1997 Jul	1997	To test if hypoglycemia unawareness and impaired counterregulation are reversible
Fanelli CG.; Dence CS.; Markham J.; Videen TO.; Paramore DS.; Cryer PE.; Powers WJ.<br>Blood-to-brain glucose transport and cerebral glucose metabolism are not reduced in poorly controlled type 1 diabetes.<br>Diabetes Sep 1998;47(9):1444-50, 1998 Sep	1998	To test the hypothesis that blood-to-brain glucose transport is reduced in poorly controlled type 1 diabetes
Fanelli C.; Calderone S.; Epifano L.; De Vincenzo A.; Modarelli F.; Pampanelli S.; Perriello G.; De Feo P.; Brunetti P.; Gerich JE.<br>Demonstration of a critical role for free fatty acids in mediating counterregulatory stimulation of gluconeogenesis and suppression of glucose utilization in humans.<br>The Journal of clinical investigation Oct 1993;92(4):1617-22, 1993 Oct	1993	To test the hypothesis that FFA mediate changes in glucose metabolism
Fabris C.; Sparacino G.; Sejling AS.; Goljahani A.; Duun-Henriksen J.; Remvig LS.; Juhl CB.; Cobelli C.	2014	Investigate if properties of the EEG signal measured by nonlinear

Hypoglycemia-related electroencephalogram changes assessed by multiscale entropy.<br>Diabetes technology & therapeutics Oct 2014;16(10):688-94<br>2014 Oct		entropy-based algorithms are altered in a significant manner when a state of hypoglycemia is entered
Fanelli CG.; Epifano L.; Rambotti AM.; Pampanelli S.; Di Vincenzo A.; Modarelli F.; Lepore M.; Annibale B.; Ciofetta M.; Bottini P.<br>Meticulous prevention of hypoglycemia normalizes the glycemic thresholds and magnitude of most of neuroendocrine responses to, symptoms of, and cognitive function during hypoglycemia in intensively treated patients with short-term IDDM.<br>Diabetes Nov 1993;42(11):1683-9, 1993 Nov	1993	To test the hypothesis that hypoglycemia unawareness is largely secondary to recurrent therapeutic hypoglycemia in IDDM
Fanelli C.; Pampanelli S.; Epifano L.; Rambotti AM.; Ciofetta M.; Modarelli F.; Di Vincenzo A.; Annibale B.; Lepore M.; Lalli C.<br>Relative roles of insulin and hypoglycaemia on induction of neuroendocrine responses to, symptoms of, and deterioration of cognitive function in hypoglycaemia in male and female humans.<br>Diabetologia Aug 1994;37(8):797-807, 1994 Aug	1994	To assess the relative roles of insulin and hypoglycaemia on induction of neuroendocrine responses, symptoms and deterioration of cognitive function
Fanelli C.; Pampanelli S.; Calderone S.; Lepore M.; Annibale B.; Compagnucci P.; Brunetti P.; Bolli GB.<br>Effects of recent, short-term hyperglycemia on responses to hypoglycemia in humans. Relevance to the pathogenesis of hypoglycemia unawareness and hyperglycemia-induced insulin resistance.<br>Diabetes May 1995;44(5):513-9, 1995 May	1995	To assess whether short-term, antecedent hyperglycemia exerts effects opposite to those observed after acute hypoglycemia
Fanelli CG.; Paramore DS.; Hershey T.; Terkamp C.; Ovalle F.; Craft S.; Cryer PE.<br>Impact of nocturnal hypoglycemia on hypoglycemic cognitive dysfunction in type 1 diabetes.<br>Diabetes Dec 1998;47(12):1920-7, 1998 Dec	1998	The impact of nocturnal hypoglycaemia in cognitive function
Fanelli C.; Pampanelli S.; Epifano L.; Rambotti AM.; Di Vincenzo A.; Modarelli F.; Ciofetta M.; Lepore M.; Annibale B.; Torlone E.<br>Long-term recovery from unawareness, deficient counterregulation and lack of cognitive dysfunction during hypoglycaemia, following institution of rational, intensive insulin therapy in IDDM.<br>Diabetologia Dec 1994;37(12):1265-76, 1994 Dec	1994	To test if hypoglycaemia unawareness is reversible as long as hypoglycaemia is meticulously prevented and that intensive insulin therapy can maintain long-term near normoglycaemia.
Farngren J.; Persson M.; Schweizer A.; Foley JE.; Ahrén B.<br>Vildagliptin reduces glucagon during hyperglycemia and sustains glucagon counterregulation during hypoglycemia in type 1 diabetes.<br>The Journal of clinical endocrinology and metabolism Oct 2012;97(10):3799-806, 2012 Oct	2012	To study whether vildagliptin also improves α-cell function in type 1 diabetes.
Farngren J.; Persson M.; Schweizer A.; Foley JE.; Ahrén B.<br>Glucagon dynamics during hypoglycaemia and food-re-challenge following treatment with vildagliptin in insulin-treated patients with type 2 diabetes.<br>Diabetes, obesity & metabolism Sep 2014;16(9):812-8, 2014 Sep	2014	To determine the effects of dipeptidyl peptidase-4 (DPP-4) inhibition on glucagon dynamics in patients with insulin-treated type 2 diabetes (T2D)
Farngren J.; Persson M.; Ahrén B.<br>Effect of the GLP-1 Receptor Agonist Lixisenatide on Counterregulatory Responses to Hypoglycemia in Subjects With Insulin-Treated Type 2 Diabetes.<br>Diabetes care Feb 2016;39(2):242-9, 2016 Feb	2016	Effect of the glucagon-like peptide 1 receptor agonist lixise natide on the hormonal counterregul atory responses to

		insulin-<br>induced hypoglycemia
Farngren J.; Persson M.; Ahrén B.<br>Effects on the glucagon response to hypoglycaemia during DPP-4 inhibition in elderly subjects with type 2 diabetes: A randomized, placebo-controlled study.<br>Diabetes, obesity & metabolism 08 2018;20(8):1911-1920, 2018 08	2018	To test whether DPP-4 inhibition affects the glucagon response to hypoglycaemia in the elderly
Flanagan DE.; Evans ML.; Monsod TP.; Rife F.; Heptulla RA.; Tamborlane WV.; Sherwin RS.<br>The influence of insulin on circulating ghrelin.<br>American journal of physiology. Endocrinology and metabolism Feb 2003;284(2):E313-6, 2003 Feb	2003	To assess the influence of insulin on circulating ghrelin
Fourest-Fontecave S.; Adamson U.; Lins PE.; Ekblom B.; Sandahl C.; Strand L.<br>Mental alertness in response to hypoglycaemia in normal man: the effect of 12 hours and 72 hours of fasting.<br>Diabetes & metabolisme ;13(4):405-10, 1987	1987	To study the influence of hypo and starvation
Frandsen CS.; Dejgaard TF.; Andersen HU.; Holst JJ.; Hartmann B.; Thorsteinsson B.; Madsbad S.<br>Liraglutide as adjunct to insulin treatment in type 1 diabetes does not interfere with glycaemic recovery or gastric emptying rate during hypoglycaemia: A randomized, placebo-controlled, double-blind, parallel-group study.<br>Diabetes, obesity & metabolism 06 2017;19(6):773-782, 2017 06	2017	the effect of the GLP-1RA liraglutide on counterregulatory responses and GE rate during hypoglycaemia in persons with T1D
Fredheim S.; Foli-Andersen P.; Laerkholm G.; Svensson J.; Juhl CB.; Olsen B.; Pilgaard K.; Johannesen J.<br>Adrenaline and cortisol levels are lower during nighttime than daytime hypoglycaemia in children with type 1 diabetes.<br>Acta paediatrica (Oslo, Norway : 1992) Oct 2018;107(10):1759-1765<br>2018 Oct	2018	To investigate children's counter regulatory hormone profiles during a hyperinsulinaemic hypoglycaemic clamp procedure at day and night
Friedrich A.; Ludwig AK.; Jauch-Chara K.; Loebig M.; Rudolf S.; Tauchert S.; Diedrich K.; Schweiger U.; Oltmanns KM.<br>Oral contraception enhances growth hormone responsiveness to hyper- and hypoglycaemia.<br>Diabetic medicine : a journal of the British Diabetic Association Mar 2012;29(3):345-50, 2012 Mar	2012	To test if<br>serum growth hormone concentrations display a differential response on glycaemic variations in healthy women using oral contraceptive s and those not using contraceptives
Fritsche A.; Stumvoll M.; Grüb M.; Sieslack S.; Renn W.; Schmülling RM.; Häring HU.; Gerich JE.<br>Effect of hypoglycemia on beta-adrenergic sensitivity in normal and type 1 diabetic subjects.<br>Diabetes care Sep 1998;21(9):1505-10, 1998 Sep	1998	To assess the potential role of reduced tissue sensitivity to catecholamines in the pathogenesis of hypoglycemia unawareness in patients with type 1 diabetes
Fruehwald-Schultes B.; Kern W.; Oltmanns KM.; Sopke S.; Toschek B.; Born J.; Fehm HL.; Peters A.<br>Metformin does not adversely affect hormonal and symptomatic responses to recurrent hypoglycemia.<br>The Journal of clinical endocrinology and metabolism Sep 2001;86(9):4187-92, 2001 Sep	2001	The effects of metformin on hormonal and symptomatic responses to hypoglycemia
Fruehwald-Schultes B.; Kern W.; Deininger E.; Wellhoener P.; Kerner W.; Born J.; Fehm HL.; Peters A.	1999	To elucidate the role of insulin in the mechanism responsible for the antecedent

Protective effect of insulin against hypoglycemia-associated counterregulatory failure.<br>The Journal of clinical endocrinology and metabolism May 1999;84(5):1551-7, 1999 May		hypoglycemia causing subsequent counterregulatory failure
Fruehwald-Schultes B.; Born J.; Kern W.; Peters A.; Fehm HL.<br>Adaptation of cognitive function to hypoglycemia in healthy men.<br>Diabetes care Aug 2000;23(8):1059-66, 2000 Aug	2000	To test the effect of antecedent hypoglycemia on hypoglycemic counterregulation and symptoms
Fruehwald-Schultes B.; Kern W.; Bong W.; Wellhoener P.; Kerner W.; Born J.; Fehm HL.; Peters A.<br>Supraphysiological hyperinsulinemia acutely increases hypothalamic-pituitary-adrenal secretory activity in humans.<br>The Journal of clinical endocrinology and metabolism Sep 1999;84(9):3041-6, 1999 Sep	1999	Whether insulin could cause HPA axis ac tivation and, if so, whether this insulin action may arise at the adrenal level or at a central
Fruehwald-Schultes B.; Kern W.; Dantz D.; Born J.; Fehm HL.; Peters A.<br>Preserved hypothermic response to hypoglycemia after antecedent hypoglycemia.<br>Metabolism: clinical and experimental Jun 2000;49(6):794-8, 2000 Jun	2000	The effects of antecedent hypoglycemia on the subsequent hypothermic response
Frystyk J.; Hussain M.; Skjaerbaek C.; Schmitz O.; Christiansen JS.; Froesch ER.; Orskov H.<br>Serum free IGF-I during a hyperinsulinemic clamp following 3 days of administration of IGF-I vs. saline.<br>The American journal of physiology Sep 1997;273(3 Pt 1):E507-13<br>1997 Sep	1997	To compare the effect of subcutanaous IGF-10 during a euglycaemic- and hypo glycaemic clamp
Færch LH.; Thorsteinsson B.; Tarnow L.; Holst JJ.; Kjær T.; Kanters J.; Larroude C.; Dela F.; Pedersen-Bjergaard U.<br>Effects of angiotensin II receptor blockade on cerebral, cardiovascular, counter-regulatory, and symptomatic responses during hypoglycaemia in patients with type 1 diabetes.<br>Journal of the renin-angiotensin-aldosterone system : JRAAS Dec 2015;16(4):1036-45, 2015 Dec	2015	To investigate whether angiotensin II receptor blockade improves cerebral and cardiovascular function during hypoglycaemia
Gabriely I.; Shamoon H.<br>Fructose normalizes specific counterregulatory responses to hypoglycemia in patients with type 1 diabetes.<br>Diabetes Mar 2005;54(3):609-16, 2005 Mar	2005	The effect of fructose to counterregulatory hormone responses to hypoglycaemia
Gabriely I.; Hawkins M.; Vilcu C.; Rossetti L.; Shamoon H.<br>Fructose amplifies counterregulatory responses to hypoglycemia in humans.<br>Diabetes Apr 2002;51(4):893-900, 2002 Apr	2002	Examined the effects of an acute infusion of fructose on hypoglycemia counterregulation
Gabriely I.; Wozniak R.; Hawkins M.; Shamoon H.<br>Troglitazone amplifies counterregulatory responses to hypoglycemia in nondiabetic subjects.<br>The Journal of clinical endocrinology and metabolism Feb 2001;86(2):521-8, 2001 Feb	2001	Evaluated the effects of a short course of troglitazone on counterregulatory hormones in response to mild hypoglycemia
Gallen IW.; Macdonald IA.<br>Effects of blood glucose concentration on thermogenesis and glucose disposal during hyperinsulinaemia.<br>Clinical science (London, England : 1979) Sep 1990;79(3):279-85<br>1990 Sep	1990	The effect of variation in the blood glucose concentration on the thermogenic and cardiovascular responses
Galassetti P.; Mann S.; Tate D.; Neill RA.; Costa F.; Wasserman DH.; Davis SN.<br>Effects of antecedent prolonged exercise on subsequent counterregulatory responses to hypoglycemia.	2001	To investigate if prior exercise blunt counterregulatory responses to

American journal of physiology. Endocrinology and metabolism Jun 2001;280(6):E908-17, 2001 Jun		subsequent hypoglycemia
Galassetti P.; Tate D.; Neill RA.; Morrey S.; Wasserman DH.; Davis SN.<br>Effect of antecedent hypoglycemia on counterregulatory responses to subsequent euglycemic exercise in type 1 diabetes.<br>Diabetes Jul 2003;52(7):1761-9, 2003 Jul	2003	To examine if prior hypoglycemia results in acute counterregulatory failure during subsequent exercise in type 1 diabetes
Geddes J.; Deary IJ.; Frier BM.<br>Effects of acute insulin-induced hypoglycaemia on psychomotor function: people with type 1 diabetes are less affected than non-diabetic adults.<br>Diabetologia Oct 2008;51(10):1814-21, 2008 Oct	2008	The effects of acute ins ulin induced hypoglycaemia on psyc homotor function
Gejl M.; Lerche S.; Egefjord L.; Brock B.; Møller N.; Vang K.; Rodell AB.; Bibby BM.; Holst JJ.; Rungby J.; Gjedde A.<br>Glucagon-like peptide-1 (GLP-1) raises blood-brain glucose transfer capacity and hexokinase activity in human brain.<br>Frontiers in neuroenergetics 2013;5():2 2013	2013	To determine the effect of GLP-1 on hypoglycemia
Gejl M.; Gjedde A.; Brock B.; Møller A.; van Duinkerken E.; Haahr HL.; Hansen CT.; Chu PL.; Stender-Petersen KL.; Rungby J.<br>Effects of hypoglycaemia on working memory and regional cerebral blood flow in type 1 diabetes: a randomised, crossover trial.<br>Diabetologia 03 2018;61(3):551-561, 2018 03	2018	To compare cognitive functioning and associated brain activation patterns during hypoglycaemia and euglycaemia
George E.; Harris N.; Bedford C.; Macdonald IA.; Hardisty CA.; Heller SR.<br>Prolonged but partial impairment of the hypoglycaemic physiological response following short-term hypoglycaemia in normal subjects.<br>Diabetologia Oct 1995;38(10):1183-90, 1995 Oct	1995	To test the effect on endocrine and symptomatic responses to hypoglycaemia 18-24 h after antecedent hypoglycaemia
George E.; Marques JL.; Harris ND.; Macdonald IA.; Hardisty CA.; Heller SR.<br>Preservation of physiological responses to hypoglycemia 2 days after antecedent hypoglycemia in patients with IDDM.<br>Diabetes care Aug 1997;20(8):1293-8 1997 Aug	1997	To assess the effects of short-term antecedent hypoglycemia on responses to further hypoglycemia 2 days later in patients with IDDM
George PS.; McCrimmon RJ.<br>Saxagliptin co-therapy in C-peptide negative Type 1 diabetes does not improve counter-regulatory responses to hypoglycaemia.<br>Diabetic medicine : a journal of the British Diabetic Association 09 2016;33(9):1283-90,2016 09	2016	To assess that dipeptidyl peptidase-4 inhibition in C-peptide negative Type 1 diabetes would reduce glucose variability and exposure to hypoglycaemia
Gogitidze Joy N.; Hedrington MS.; Briscoe VJ.; Tate DB.; Ertl AC.; Davis SN.<br>Effects of acute hypoglycemia on inflammatory and pro-atherothrombotic biomarkers in individuals with type 1 diabetes and healthy individuals.<br>Diabetes care Jul 2010;33(7):1529-35, 2010 Jul	2010	To determine whether 2 h of moderate clamped hypoglycemia could decrease fibrinolytic balance and activate pro-atherothrombotic mechanisms in individuals
Gold AE.; MacLeod KM.; Frier BM.; Deary IJ.<br>Changes in mood during acute hypoglycemia in healthy participants.<br>Journal of personality and social psychology Mar 1995;68(3):498-504 1995 Mar	1995	To investigate the mood changes during acute hypoglycemia
Gold AE.; MacLeod KM.; Deary IJ.; Frier BM.<br>Hypoglycemia-induced cognitive dysfunction in diabetes mellitus: effect of hypoglycemia unawareness.	1995	To assess and compare the degree of cognitive dysfunction

Physiology & behavior Sep 1995;58(3):501-11, 1995 Sep		experienced by insulin-dependent diabetic patients
Gosmanov NR.; Szoke E.; Israelian Z.; Smith T.; Cryer PE.; Gerich JE.; Meyer C.<br>Role of the decrement in intraislet insulin for the glucagon response to hypoglycemia in humans.<br>Diabetes care May 2005;28(5):1124-31, 2005 May	2005	To study if somatostatin affects the glucagon secretion
Grøfte T.; Wolthers T.; Jørgensen JO.; Poulsen PL.; Vilstrup H.; Møller N.<br>Hepatic amino- to urea-N clearance and forearm amino-N exchange during hypoglycemic and euglycemic hyperinsulinemia in normal man.<br>Journal of hepatology May 1999;30(5):819-25, 1999 May	1999	The effect of hypoglycaemia on hepatic amino nitrogen conversion in relation<br>to muscle amino nitrogen flux
Graveling AJ.; Deary IJ.; Frier BM.<br>Acute hypoglycemia impairs executive cognitive function in adults with and without type 1 diabetes.<br>Diabetes care Oct 2013;36(10):3240-6 2013 Oct	2013	The effect of acute hypoglycemia on executive function in adults with and without diabetes
Grant PJ.; Stickland MH.; Bristow AF.; Clarke RF.; Wales JK.<br>Insulin increases plasma somatomedin C (IGF-1) concentrations in adult type 1 diabetic patients.<br>Diabetic medicine : a journal of the British Diabetic Association Apr 1989;6(3):245-8, 1989 Apr	1989	The effect of insulin in the control of somatomedin release
Gustavson SM.; Sandoval DA.; Ertl AC.; Bao S.; Raj SR.; Davis SN.<br>Stimulation of both type I and type II corticosteroid receptors blunts counterregulatory responses to subsequent hypoglycemia in healthy man.<br>American journal of physiology. Endocrinology and metabolism Mar 2008;294(3):E506-12, 2008 Mar	2008	To determine whether prior activation<br>of type I corticosteroid or type II corticosteroid receptors blunts counte rregulatory responses t o subsequent hypoglyc emia
Hagström-Toft E.; Enoksson S.; Moberg E.; Bolinder J.; Arner P.<br>beta-Adrenergic regulation of lipolysis and blood flow in human skeletal muscle in vivo.<br>The American journal of physiology Dec 1998;275(6 Pt 1):E909-16, 1998 Dec	1998	To investiagte β-adrenergic regulation of lipolysis and blood flow was investigated
Hedrington MS.; Farmerie S.; Ertl AC.; Wang Z.; Tate DB.; Davis SN.<br>Effects of antecedent GABAA activation with alprazolam on counterregulatory responses to hypoglycemia in healthy humans.<br>Diabetes Apr 2010;59(4):1074-81, 2010 Apr	2010	To determine<br>the effects of prior GABA(A) activation usin g the<br>benzodiazepine alprazo lam on the neuroendocrine and autonomic nervous system (ANS) and metabolic counterregul atory responses
Hedrington MS.; Tate DB.; Younk LM.; Davis SN.<br>Effects of Antecedent GABA A Receptor Activation on Counterregulatory Responses to Exercise in Healthy Man.<br>Diabetes Sep 2015;64(9):3253-61, 2015 Sep	2015	To test the hypothesis that antecedent pharmacologic activation of GABA A receptors with alprazolam can result in neuroendocrine, ANS, and/or metabolic counterregulatory failure during next-day moderate exercise in healthy humans

Heise T.; Heinemann L.; Heller S.; Weyer C.; Wang Y.; Strobel S.; Kolterman O.; Maggs D.<br>Effect of pramlintide on symptom, catecholamine, and glucagon responses to hypoglycemia in healthy subjects.<br>Metabolism: clinical and experimental Sep 2004;53(9):1227-32, 2004 Sep	2004	To test the effect of pramlintide<br>on symptom, catechola mine,<br>and glucagon responses
Hermanns N.; Plate M.; Kulzer B.; Fischer B.; Linn T.; Bretzel R.; Haak T.<br>Effect of experimentally induced hypoglycemia and different insulin levels on feelings of hunger in type 1 diabetic patients.<br>Experimental and clinical endocrinology & diabetes : official journal, German Society of Endocrinology [and] German Diabetes Association May 2008;116(5):255-61, 2008 May	2008	Investigate the impacts of experimentally induced hypoglycemia and different insulin infusion rates on feelings of hunger
Hermanns N.; Kubiak T.; Kulzer B.; Haak T.<br>Emotional changes during experimentally induced hypoglycaemia in type 1 diabetes.<br>Biological psychology Apr 2003;63(1):15-44, 2003 Apr	2003	To determine emotional changes during experimentally induced hypoglycaemia
Heptulla RA.; Tamborlane WV.; Ma TY.; Rife F.; Sherwin RS.<br>Oral glucose augments the counterregulatory hormone response during insulin-induced hypoglycemia in humans.<br>The Journal of clinical endocrinology and metabolism Feb 2001;86(2):645-8, 2001 Feb	2001	To evaluate the effect of raising portal, but not peripheral, glucose levels during insulin-induced hypoglycemia
Hinshaw L.; Mallad A.; Dalla Man C.; Basu R.; Cobelli C.; Carter RE.; Kudva YC.; Basu A.<br>Glucagon sensitivity and clearance in type 1 diabetes: insights from in vivo and in silico experiments.<br>American journal of physiology. Endocrinology and metabolism Sep 2015;309(5):E474-86, 2015 Sep	2015	The effect of glucagon sensitivity during hypoglycemia vs. euglycemia
Hirsch IB.; Heller SR.; Cryer PE.<br>Increased symptoms of hypoglycaemia in the standing position in insulin-dependent diabetes mellitus.<br>Clinical science (London, England : 1979) Jun 1991;80(6):583-6, 1991 Jun	1991	To determine the effect of position on the amount of symptoms of hypoglycaemia
Hirsch IB.; Boyle PJ.; Craft S.; Cryer PE.<br>Higher glycemic thresholds for symptoms during beta-adrenergic blockade in IDDM.<br>Diabetes Sep 1991;40(9):1177-86, 1991 Sep	1991	The effect of nonselective beta adrenergic blockade on hypoglycemia unawareness
Hoffman RP.; Sinkey CA.; Anderson EA.<br>Hypoglycemic symptom variation is related to epinephrine and not peripheral muscle sympathetic nerve response.<br>Journal of diabetes and its complications ;11(1):15-20	1997	The effect of nonselective beta adrenergic blockade on hypoglycemic symptoms
Hoffman RP.; Sinkey CA.; Anderson EA.<br>Hypoglycemia increases muscle sympathetic nerve activity in IDDM and control subjects.<br>Diabetes care Jul 1994;17(7):673-80, 1994 Jul	1994	To assess the relationship between the increase in adrenomedullary catecholamine secretion and the sympathetic response
Hoffman RP.; Sinkey CA.; Dopp JM.; Phillips BG.<br>Systemic and local adrenergic regulation of muscle glucose utilization during hypoglycemia in healthy subjects.<br>Diabetes Mar 2002;51(3):734-42, 2002 Mar	2002	To distinguish between the local and systemic effects of hypoglycemic adrenergic counterregulation on FGU and metabolism
Hoffman RP.<br>Antecedent hypoglycemia does not alter increased epinephrine-induced lipolysis in type 1 diabetes mellitus.<br>Metabolism: clinical and experimental Mar 2006;55(3):371-80, 2006 Mar	2006	To determine whether type 1 diabetic subjects have<br>increased metabolic response to epinephrin e

Hompesch M.; Jones-Leone A.; Carr MC.; Matthews J.; Zhi H.; Young M.; Morrow L.; Reinhardt RR.<br>Albiglutide does not impair the counter-regulatory hormone response to hypoglycaemia: a randomized, double-blind, placebo-controlled, stepped glucose clamp study in subjects with type 2 diabetes mellitus.<br>Diabetes, obesity & metabolism Jan 2015;17(1):82-90, 2015 Jan	2015	To determine if the glucagon-like peptide-1 (GLP-1) receptor agonist albiglutide, once weekly, impairs counter-regulatory responses during hypoglycaemia
Hussain MA.; Schmitz O.; Christiansen JS.; Christensen NJ.; Alberti KG.; Froesch ER.<br>IGF-I alters skeletal muscle substrate metabolism and blunts recovery from insulin-induced hypoglycemia.<br>The American journal of physiology Apr 1996;270(4 Pt 1):E545-51, 1996 Apr	1996	The effect of IGF-I on skeletal muscle substrate metabolisme
Israelian Z.; Szoke E.; Woerle J.; Bokhari S.; Schorr M.; Schwenke DC.; Cryer PE.; Gerich JE.; Meyer C.<br>Multiple defects in counterregulation of hypoglycemia in modestly advanced type 2 diabetes mellitus.<br>Metabolism: clinical and experimental May 2006;55(5):593-8, 2006 May	2006	The effect of a 2-hour hyperinsulinemic hypog lycemic clamp on hormonal responses including rates of insulin secretion
Israelian Z.; Gosmanov NR.; Szoke E.; Schorr M.; Bokhari S.; Cryer PE.; Gerich JE.; Meyer C.<br>Increasing the decrement in insulin secretion improves glucagon responses to hypoglycemia in advanced type 2 diabetes.<br>Diabetes care Nov 2005;28(11):2691-6, 2005 Nov	2005	To investigate of insulin secretion on glucagon responses to hypoglycaemia in DM2
Inkster BE.; Zammitt NN.; Ritchie SJ.; Deary IJ.; Morrison I.; Frier BM.<br>Effects of Sleep Deprivation on Hypoglycemia-Induced Cognitive Impairment and Recovery in Adults With Type 1 Diabetes.<br>Diabetes care 05 2016;39(5):750-6, 2016 05	2016	To investigate the effects of sleep deprivation on hypoglycemia-induced cognitive impairment and recovery
Janssen MM.; Snoek FJ.; Heine RJ.<br>Assessing impaired hypoglycemia awareness in type 1 diabetes: agreement of self-report but not of field study data with the autonomic symptom threshold during experimental hypoglycemia.<br>Diabetes care Apr 2000;23(4):529-32<br>2000 Apr	2000	To determine the agreement of two noninvasive methods, a self-report and a field study method, for the assessment of impaired hypoglycemia awareness
Janssen MM.; Snoek FJ.; Masurel N.; Hoogma RP.; Devillé WL.; Popp-Snijders C.; Heine RJ.<br>Optimized basal-bolus therapy using a fixed mixture of 75% lispro and 25% NPL insulin in type 1 diabetes patients: no favorable effects on glycemic control, physiological responses to hypoglycemia, well-being, or treatment satisfaction.<br>Diabetes care May 2000;23(5):629-33<br>2000 May	2000	To investigate the effects of a multiple injection regimen with a mixture of 75%lispro and 25% intermediate-acting insulin
Jennum P.; Stender-Petersen K.; Rabøl R.; Jørgensen NR.; Chu PL.; Madsbad S.<br>The Impact of Nocturnal Hypoglycemia on Sleep in Subjects With Type 2 Diabetes.<br>Diabetes care Nov 2015;38(11):2151-7<br>2015 Nov	2015	To investigate the impact of nocturnal hypoglycemia on sleep patterns and counterregulatory hormones
Jiang LL.; Wang SQ.; Ding B.; Zhu J.; Jing T.; Ye L.; Lee KO.; Wu JD.; Ma JH.<br>The effects of add-on exenatide to insulin on glycemic variability and hypoglycemia in patients with type 1 diabetes mellitus.<br>Journal of endocrinological investigation May 2018;41(5):539-547<br>2018 May	2018	To investigate the effect of add-on exenatide to insulin on glycemic excursion and the counter-regulatory hormone in response to hypoglycemia in

		patients with type 1 diabetes mellitus
Jones TW.; Borg WP.; Borg MA.; Boulware SD.; McCarthy G.; Silver D.; Tamborlane WV.; Sherwin RS.<br>Resistance to neuroglycopenia: an adaptive response during intensive insulin treatment of diabetes.<br>The Journal of clinical endocrinology and metabolism Jun 1997;82(6):1713-8<br>1997 Jun	1997	To determine whether counterregulation and awareness of hypoglycaemia are associated with an alteration in the susceptibility of the brain to mild hypoglycemia
Jones TW.; Borg WP.; Boulware SD.; McCarthy G.; Sherwin RS.; Tamborlane WV.<br>Enhanced adrenomedullary response and increased susceptibility to neuroglycopenia: mechanisms underlying the adverse effects of sugar ingestion in healthy children.<br>The Journal of pediatrics Feb 1995;126(2):171-7, 1995 Feb	1995	Comparison of the metabolic, hormonal, and symptomatic responses of healthy children and healthy young adults with a standardized, large glucose load.
Jones TW.; Boulware SD.; Kraemer DT.; Caprio S.; Sherwin RS.; Tamborlane WV.<br>Independent effects of youth and poor diabetes control on responses to hypoglycemia in children.<br>Diabetes Mar 1991;40(3):358-63, 1991 Mar	1991	To evaluate the effects of childhood and poorly controlled insulin-dependent diabetes mellitus
Jones TW.; Porter P.; Sherwin RS.; Davis EA.; O'Leary P.; Frazer F.; Byrne G.; Stick S.; Tamborlane WV.<br>Decreased epinephrine responses to hypoglycemia during sleep.<br>The New England journal of medicine Jun 1998;338(23):1657-62, 1998 Jun	1998	The effect of epinephrine responses to hypoglycemia during sleep
Jones TW.; McCarthy G.; Tamborlane WV.; Caprio S.; Roessler E.; Kraemer D.; Starick-Zych K.; Allison T.; Boulware SD.; Sherwin RS.<br>Mild hypoglycemia and impairment of brain stem and cortical evoked potentials in healthy subjects.<br>Diabetes Dec 1990;39(12):1550-5, 1990 Dec	1990	To evaluate the impact of mild hypoglycemia on CNS function in healthy adults
Joy NG.; Perkins JM.; Mikeladze M.; Younk L.; Tate DB.; Davis SN.<br>Comparative effects of acute hypoglycemia and hyperglycemia on pro-atherothrombotic biomarkers and endothelial function in non-diabetic humans.<br>Journal of diabetes and its complications ;30(7):1275-81	2016	The comparative effects of acute moderate hyperglycemia and hypoglycemia on in vivo endothelial function together
Joy NG.; Tate DB.; Younk LM.; Davis SN.<br>Effects of Acute and Antecedent Hypoglycemia on Endothelial Function and Markers of Atherothrombotic Balance in Healthy Humans.<br>Diabetes Jul 2015;64(7):2571-80, 2015 Jul	2015	To determine the effects of single and repeated episodes of clamped hypoglycemia
Joy NG.; Tate DB.; Davis SN.<br>Counterregulatory responses to hypoglycemia differ between glimepiride and glyburide in non diabetic individuals.<br>Metabolism: clinical and experimental Jun 2015;64(6):729-37, 2015 Jun	2015	To determine whether physiologic differences in counterregulatory neuroendocrine and metabolic mechanisms during hypoglycemia provide a basis for the observed clinical differences between glimepiride and glyburide
Kanc K.; Janssen MM.; Keulen ET.; Jacobs MA.; Popp-Snijders C.; Snoek FJ.; Heine RJ.<br>Substitution of night-time continuous subcutaneous insulin infusion therapy for bedtime NPH insulin in a multiple injection regimen improves	1998	Assessed the influence of a more stable nocturnal blood glucose control on the counterregulatory hormonal responses

counterregulatory hormonal responses and warning symptoms of hypoglycaemia in IDDM.<br>Diabetologia Mar 1998;41(3):322-9, 1998 Mar		and symptoms of hypoglycaemia.
Kempe K.; Price D.; Ellison J.; Marhoul J.; Morrow L.; Win K.; Kuschma K.; Hompesch M.<br>Capillary and venous blood glucose concentrations measured during intravenous insulin and glucose infusion: a comparison of steady and dynamic states.<br>Diabetes technology & therapeutics Oct 2009;11(10):669-74, 2009 Oct	2009	To compared capillary and venous BG across a range of glucose concentrations and under conditions of rapid change in BG
Kendall DM.; Teuscher AU.; Robertson RP.<br>Defective glucagon secretion during sustained hypoglycemia following successful islet allo- and autotransplantation in humans.<br>Diabetes Jan 1997;46(1):23-7<br>1997 Jan	1997	To determine whether defective glucagon secretion can be corrected by successful intrahepatic islet transplantation
Kerr D.; Macdonald IA.; Tattersall RB.<br>Adaptation to mild hypoglycaemia in normal subjects despite sustained increases in counter-regulatory hormones.<br>Diabetologia Apr 1989;32(4):249-54 1989 Apr'	1989	The effects of adaptation to mild hypoglycaemia
Kerr D.; Macdonald IA.; Tattersall RB.<br>Patients with type 1 diabetes adapt acutely to sustained mild hypoglycaemia.<br>Diabetic medicine : a journal of the British Diabetic Association ;8(2):123-8	1991	The effect of sustained mild hypoglycaemia on symptoms of hypoglycaemia
Kerr D.; MacDonald IA.; Tattersall RB.<br>Influence of duration of hypoglycemia on the hormonal counterregulatory response in normal subjects.<br>The Journal of clinical endocrinology and metabolism Jun 1989;68(6):1118-22, 1989 Jun	1989	To investigate the counterregulatory response to recurrent and prolonged mild hypoglycemia in normal women
Kerr D.; Macdonald IA.; Heller SR.; Tattersall RB.<br>Alcohol causes hypoglycaemic unawareness in healthy volunteers and patients with type 1 (insulin-dependent) diabetes.<br>Diabetologia Apr 1990;33(4):216-21, 1990 Apr	1990	To examine the effect of both hypoglycaemia and alcohol consumption on cognitive function
Kerr D.; Reza M.; Smith N.; Leatherdale BA.<br>Importance of insulin in subjective, cognitive, and hormonal responses to hypoglycemia in patients with IDDM.<br>Diabetes Aug 1991;40(8):1057-62, 1991 Aug	1991	To examine whether insulin has an effect, independent of blood glucose, on the subjective, cognitive, and hormonal responses to hypoglycemia
Kerr D.; Tamborlane WV.; Rife F.; Sherwin RS.<br>Effect of insulin-like growth factor-1 on the responses to and recognition of hypoglycemia in humans. A comparison with insulin.<br>The Journal of clinical investigation Jan 1993;91(1):141-7, 1993 Jan	1993	To investigate the effect of insulin-like growth factor-1 on the responses to and recognition of hypoglycemia in humans
Kerr D.; Stanley JC.; Barron M.; Thomas R.; Leatherdale BA.; Pickard J.<br>Symmetry of cerebral blood flow and cognitive responses to hypoglycaemia in humans.<br>Diabetologia Jan 1993;36(1):73-8, 1993 Jan	1993	To determine differences in the physiological responses to hypoglycaemia between the cerebral hemispheres
Kerr D.; Diamond MP.; Tamborlane WV.; Kerr S.; Sherwin RS.<br>Influence of counterregulatory hormones, independently of hypoglycaemia, on cognitive function, warning symptoms and glucose kinetics.	1993	To assess the influence of counterregulatory hormones, independently of neuroglycopenia, on

Clinical science (London, England : 1979) Aug 1993;85(2):197-202, 1993 Aug		higher cerebral (cognitive) function
Kerr D.; Sherwin RS.; Pavalkis F.; Fayad PB.; Sikorski L.; Rife F.; Tamborlane WV.; During MJ.<br>Effect of caffeine on the recognition of and responses to hypoglycemia in humans.<br>Annals of internal medicine Oct 1993;119(8):799-804, 1993 Oct	1993	To test whether two effects of acute caffeine ingestion decrease in cerebral blood flow and increase in brain glucose use alter the recognition of and physiologic responses to hypoglycemia.
Kerr D.; Cheyne E.; Thomas P.; Sherwin R.<br>Influence of acute alcohol ingestion on the hormonal responses to modest hypoglycaemia in patients with Type 1 diabetes.<br>Diabetic medicine : a journal of the British Diabetic Association Mar 2007;24(3):312-6, 2007 Mar	2007	To investigate whether mild alcohol intoxication influences counterregulatory hormone responses
Khan MI.; Barlow RB.; Weinstock RS.<br>Acute hypoglycemia decreases central retinal function in the human eye.<br>Vision research Jul 2011;51(14):1623-6, 2011 Jul	2011	The effects of acute hypoglycemia on retinal function
King P.; Parkin H.; Macdonald IA.; Barber C.; Tattersall RB.<br>The effect of intravenous lactate on cerebral function during hypoglycaemia.<br>Diabetic medicine : a journal of the British Diabetic Association Jan 1997;14(1):19-28, 1997 Jan	1997	To investigate if intravenous lactate protects cerebral function during hypoglycaemia
King C.; Anderson SM.; Breton M.; Clarke WL.; Kovatchev BP.<br>Modeling of Calibration Effectiveness and Blood-to-Interstitial Glucose Dynamics as Potential Confounders of the Accuracy of Continuous Glucose Sensors during Hyperinsulinemic Clamp.<br>Journal of diabetes science and technology May 2007;1(3):317-22, 2007 May	2007	To investigate the different models of the dynamics of interstitial fluid-based continuous glucose sensors
Kinsley BT.; Widom B.; Simonson DC.<br>Differential regulation of counterregulatory hormone secretion and symptoms during hypoglycemia in IDDM. Effect of glycemic control.<br>Diabetes care Jan 1995;18(1):17-26, 1995 Jan	1995	To investigate if there was a differential effect of glycemic control on individual counterregulatory hormone responses to hypoglycemia in patients
Kinsley BT.; Simonson DC.<br>Evidence for a hypothalamic-pituitary versus adrenal cortical effect of glycemic control on counterregulatory hormone responses to hypoglycemia in insulin-dependent diabetes mellitus.<br>The Journal of clinical endocrinology and metabolism Feb 1996;81(2):684-91, 1996 Feb	1996	To determine the effect of hypothalamic-pituitary versus adrenal cortical effect of glycemic control on counterregulatory hormone responses to hypoglycemia
Kinsley BT.; Widom B.; Utzschneider K.; Simonson DC.<br>Stimulus specificity of defects in counterregulatory hormone secretion in insulin-dependent diabetes mellitus: effect of glycemic control.<br>The Journal of clinical endocrinology and metabolism Nov 1994;79(5):1383-9, 1994 Nov	1994	The effect of counterregulatory hormone responses to hypoglycemia in subjects with insulin-dependent diabetes mellitus (IDDM) in strict glycemic control
Kinsley BT.; Weinger K.; Bajaj M.; Levy CJ.; Simonson DC.; Quigley M.; Cox DJ.; Jacobson AM.<br>Blood glucose awareness training and epinephrine responses to hypoglycemia during intensive treatment in type 1 diabetes.<br>Diabetes care Jul 1999;22(7):1022-8, 1999 Jul	1999	To determine the effect of blood glucose awareness train ing (BGAT) on epinephrine and symptom responses

Klement J.; Pais I.; Strube J.; Lehnert H.; Peters A.; Hallschmid M.; Born J.<br>NMDA receptor blockade by memantine does not prevent adaptation to recurrent hypoglycaemia in healthy men.<br>Diabetes, obesity & metabolism Apr 2013;15(4):310-5, 2013 Apr	2013	To investigate the effects of counterregulatory attenuation on a basic adaptive learning process relying on synaptic long-term potentiation or depression
Klement J.; Kubera B.; Eggeling J.; Rädel C.; Wagner C.; Park SQ.; Peters A.<br>Effects of blood glucose on delay discounting, food intake and counterregulation in lean and obese men.<br>Psychoneuroendocrinology Mar 2018;89():177-184, 2018 Mar	2018	To determine if obese people – in comparison to lean men – have a relative lack of energy, especially when blood glucose levels are low
Koivikko ML.; Salmela PI.; Airaksinen KE.; Tapanainen JS.; Ruokonen A.; Mäkikallio TH.; Huikuri HV.<br>Effects of sustained insulin-induced hypoglycemia on cardiovascular autonomic regulation in type 1 diabetes.<br>Diabetes Mar 2005;54(3):744-50,2005 Mar	2005	Effects of hypoglycemia on cardiac autonomic regulation
Koehler G.; Heller S.; Korsatko S.; Roepstorff C.; Rasmussen S.; Haahr H.; Pieber TR.<br>Insulin degludec is not associated with a delayed or diminished response to hypoglycaemia compared with insulin glargine in type 1 diabetes: a double-blind randomised crossover study.<br>Diabetologia Jan 2014;57(1):40-9, 2014 Jan	2014	The acute physiological responses to hypoglycaemia with IDeg and insulin glargine (A21Gly,B31Ar g,B32Arg human insulin; IGlar) were compared
Koivikko ML.; Karsikas M.; Salmela PI.; Tapanainen JS.; Ruokonen A.; Seppänen T.; Huikuri HV.; Perkiömäki JS.<br>Effects of controlled hypoglycaemia on cardiac repolarisation in patients with type 1 diabetes.<br>Diabetologia Mar 2008;51(3):426-35, 2008 Mar	2008	The effect nocturnal hypoglycaemia on sudden death in diabetic patients
Korzon-Burakowska A.; Hopkins D.; Matyka K.; Lomas J.; Pernet A.; Macdonald I.; Amiel S.<br>Effects of glycemic control on protective responses against hypoglycemia in type 2 diabetes.<br>Diabetes care Feb 1998;21(2):283-90, 1998 Feb	1998	To determine the effects of glycemic control on the counterregulatory resp onses to hypoglycemia i n type 2 diabetes
Korsatko S.; Jensen L.; Brunner M.; Sach-Friedl S.; Tarp MD.; Holst AG.; Heller SR.; Pieber TR.<br>Effect of once-weekly semaglutide on the counterregulatory response to hypoglycaemia in people with type 2 diabetes: A randomized, placebo-controlled, double-blind, crossover trial.<br>Diabetes, obesity & metabolism Nov 2018;20(11):2565-2573, 2018 Nov	2018	To investigate the effects of semaglutide vs placebo on glucagon and other counterregulatory hormones
Kraegen EW.; Lazarus L.; Campbell LV.<br>Failure of insulin infusion during euglycemia to influence endogenous basal insulin secretion.<br>Metabolism: clinical and experimental Jun 1983;32(6):622-7<br>1983 Jun	1983	To examine whether endogenous insulin influences insulin secretion independently of blood glucose
Krentz AJ.; Morrow L.; Petersson M.; Norjavaara E.; Hompesch M.<br>Effect of exogenously administered glucagon versus spontaneous endogenous counter-regulation on glycaemic recovery from insulin-induced hypoglycaemia in patients with type 2 diabetes treated with a novel glucokinase activator, AZD1656, and metformin.<br>Diabetes, obesity & metabolism Nov 2014;16(11):1096-101, 2014 Nov	2014	To study the effect of exogenous i.m. glucagon on recovery from controlled insulin-induced hypoglycaemia in patients with type 2 diabetes treated with the novel glucokinase activator AZD1656, in

		combination with metformin
Kristensen PL.; Pedersen-Bjergaard U.; Kjær TW.; Olsen NV.; Dela F.; Holst JJ.; Faber J.; Tarnow L.; Thorsteinsson B.<br>Influence of erythropoietin on cognitive performance during experimental hypoglycemia in patients with type 1 diabetes mellitus: a randomized cross-over trial.<br>PloS one 2013;8(4):e59672, 2013	2013	To investigate the influence of erythropoietin on cognitive performance during experimental hypoglycemia
Kubera B.; Klement J.; Wagner C.; Rädel C.; Eggeling J.; Füllbrunn S.; Kaczmarek MC.; Levinsky R.; Peters A.<br>Differences in fairness and trust between lean and corpulent men.<br>International journal of obesity (2005) 11 2016;40(11):1802-1808<br>2016 11	2016	The effects of hypoglycaemia on economic decision making between lean and corpulent subjects.
Laitinen T.; Huopio H.; Vauhkonen I.; Camaro C.; Hartikainen J.; Laakso M.; Niskanen L.<br>Effects of euglycaemic and hypoglycaemic hyperinsulinaemia on sympathetic and parasympathetic regulation of haemodynamics in healthy subjects.<br>Clinical science (London, England : 1979) Sep 2003;105(3):315-22<br>2003 Sep	2003	To investigate regulatory and hemodynamic responses
Laitinen T.; Lyyra-Laitinen T.; Huopio H.; Vauhkonen I.; Halonen T.; Hartikainen J.; Niskanen L.; Laakso M.<br>Electrocardiographic alterations during hyperinsulinemic hypoglycemia in healthy subjects.<br>Annals of noninvasive electrocardiology : the official journal of the International Society for Holter and Noninvasive Electrocardiology, Inc Apr 2008;13(2):97-105, 2008 Apr	2008	We evaluated the arrhythmogenic potential of hypoglycemia
Landstedt-Hallin L.; Adamson U.; Lins PE.<br>Oral glibenclamide suppresses glucagon secretion during insulin-induced hypoglycemia in patients with type 2 diabetes.<br>The Journal of clinical endocrinology and metabolism Sep 1999;84(9):3140-5, 1999 Sep	1999	To study oral glibenclamide on glucagon secretion
Lecavalier L.; Bolli G.; Cryer P.; Gerich J.<br>Contributions of gluconeogenesis and glycogenolysis during glucose counterregulation in normal humans.<br>The American journal of physiology Jun 1989;256(6 Pt 1):E844-51<br>1989 Jun	1989	To estimate the relative contributions o f gluconeogenesis and g lycogenolysis to the increase in hepatic glucose output
Leelarathna L.; Little SA.; Walkinshaw E.; Tan HK.; Lubina-Solomon A.; Kumareswaran K.; Lane AP.; Chadwick T.; Marshall SM.; Speight J.; Flanagan D.; Heller SR.; Shaw JA.; Evans ML.<br>Restoration of self-awareness of hypoglycemia in adults with long-standing type 1 diabetes: hyperinsulinemic-hypoglycemic clamp substudy results from the HypoCOMPaSS trial.<br>Diabetes care Dec 2013;36(12):4063-70, 2013 Dec	2013	To demonstrate that by optimizing conventional management, including the use of real time continuous glucose monitoring in individuals with DM1 complicated by IAH, rigorous prevention of BH will restore awareness and reduce risk of recurrent SH
Lee SP.; Harris ND.; Robinson RT.; Davies C.; Ireland R.; Macdonald IA.; Heller SR.<br>Effect of atenolol on QTc interval lengthening during hypoglycaemia in type 1 diabetes.<br>Diabetologia Jul 2005;48(7):1269-72, 2005 Jul	2005	To investigated if beta1-blockade will prevent QTc lengthening in type 1 diabetic patients
Lee SP.; Yeoh L.; Harris ND.; Davies CM.; Robinson RT.; Leathard A.; Newman C.; Macdonald IA.; Heller SR.	2004	To study the influence of autonomic neuropathy on QTc interval lengthening

Influence of autonomic neuropathy on QTc interval lengthening during hypoglycemia in type 1 diabetes.<br>Diabetes Jun 2004;53(6):1535-42, 2004 Jun		
Lee JJ.; Khoury N.; Shackleford AM.; Nelson S.; Herrera H.; Antenor-Dorsey JA.; Semenkovich K.; Shimony JS.; Powers WJ.; Cryer PE.; Arbeláez AM.<br>Dissociation Between Hormonal Counterregulatory Responses and Cerebral Glucose Metabolism During Hypoglycemia.<br>Diabetes 12 2017;66(12):2964-2972, 2017 12	2017	To asses if recurrent hypoglycemia alters the cascade of physiological and behavioral responses that maintain euglycemia
Limberg JK.; Farni KE.; Taylor JL.; Dube S.; Basu A.; Basu R.; Wehrwein EA.; Joyner MJ.<br>Autonomic control during acute hypoglycemia in type 1 diabetes mellitus.<br>Clinical autonomic research : official journal of the Clinical Autonomic Research Society Dec 2014;24(6):275-83, 2014 Dec	2014	To investigate the effect of hypoglycaemia on measures of autonomic and cardiovascular function
Limberg JK.; Taylor JL.; Dube S.; Basu R.; Basu A.; Joyner MJ.; Wehrwein EA.<br>Role of the carotid body chemoreceptors in baroreflex control of blood pressure during hypoglycaemia in humans.<br>Experimental physiology Apr 2014;99(4):640-50, 2014 Apr	2014	To examine the role of the carotid body chemoreceptors in baroreflex control of blood pressure during hypoglycaemia
Lingenfelser T.; Steffen J.; Buettner UW.; Jakober B.<br>Changes in brainstem auditory evoked potentials during insulin-induced hypoglycaemia in type 1 diabetic patients.<br>Diabetic medicine : a journal of the British Diabetic Association Jul 1992;9(6):582-3, 1992 Jul	1992	To evaluate whether the pathways along the brainstem and auditory nerve studied by means of auditory evoked potentials are disturbed during hypoglycaemia
Lingenfelser T.; Renn W.; Sommerwerck U.; Jung MF.; Buettner UW.; Zaiser-Kaschel H.; Kaschel R.; Eggstein M.; Jakober B.<br>Compromised hormonal counterregulation, symptom awareness, and neurophysiological function after recurrent short-term episodes of insulin-induced hypoglycemia in IDDM patients.<br>Diabetes Apr 1993;42(4):610-8, 1993 Apr	1993	To test if recurrent short-term hypoglycemic episodes have an effect on hormonal counterregulation, symptom awareness, and neurophysiological function
Lingenfelser T.; Buettner UW.; Uhl H.; Renn W.; Tobis M.; Teichmann R.; Eggstein M.; Jakober B.<br>Recovery of hypoglycaemia-associated compromised cerebral function after a short interval of euglycaemia in insulin-dependent diabetic patients.<br>Electroencephalography and clinical neurophysiology May 1994;92(3):196-203, 1994 May	1994	To investigate the recovery of hypoglycaemia-associated compromised cerebral function after a short interval of euglycaemia
Liu D.; Moberg E.; Kollind M.; Lins PE.; Adamson U.<br>A high concentration of circulating insulin suppresses the glucagon response to hypoglycemia in normal man.<br>The Journal of clinical endocrinology and metabolism Nov 1991;73(5):1123-8, 1991 Nov	1991	In an attempt to clarify whether circulating insulin per se exerts an inhibitory effect on the hormonal responses to hypoglycemia
Liu DT.; Adamson UC.; Lins PE.; Kollind ME.; Moberg EA.; Andréasson K.<br>Inhibitory effect of circulating insulin on glucagon secretion during hypoglycemia in type I diabetic patients.<br>Diabetes care Jan 1992;15(1):59-65, 1992 Jan	1992	To clarify whether the circulating insulin level influences hormonal responses
Liu D.; Moberg E.; Kollind M.; Lins PE.; Adamson U.; Macdonald IA.<br>Arterial, arterialized venous, venous and capillary blood glucose measurements in normal man during hyperinsulinaemic euglycaemia and hypoglycaemia.<br>Diabetologia Mar 1992;35(3):287-90, 1992 Mar	1992	To evaluate the effectiveness of the warm-air box method on the arterialization of venous blood during euglycaemia and hypoglycaemia

Liu D.; McManus RM.; Ryan EA.<br>Improved counter-regulatory hormonal and symptomatic responses to hypoglycemia in patients with insulin-dependent diabetes mellitus after 3 months of less strict glycemic control.<br>Clinical and investigative medicine. Medecine clinique et experimentale Apr 1996;19(2):71-82, 1996 Apr	1996	Examines the effect of a period of less strict glycemic control on the defective counter-regulatory hormonal responses to and impaired awareness of hypoglycemia
Lobmann R.; Smid HG.; Pottag G.; Wagner K.; Heinze HJ.; Lehnert H.<br>Impairment and recovery of elementary cognitive function induced by hypoglycemia in type-1 diabetic patients and healthy controls.<br>The Journal of clinical endocrinology and metabolism Aug 2000;85(8):2758-66, 2000 Aug	2000	We aimed at dissecting cognitive adaptation into single components
Loud FB.; Holst JJ.; Rehfeld JF.; Christiansen J.<br>Inhibition of gastric acid secretion in humans by glucagon during euglycemia, hyperglycemia, and hypoglycemia.<br>Digestive diseases and sciences May 1988;33(5):530-4, 1988 May	1988	We have studied the relationship between blood glucose levels and the a inhibitory effect of exogenously administrated glucagon in healthy subjects
Lubow JM.; Piñón IG.; Avogaro A.; Cobelli C.; Treeson DM.; Mandeville KA.; Toffolo G.; Boyle PJ.<br>Brain oxygen utilization is unchanged by hypoglycemia in normal humans: lactate, alanine, and leucine uptake are not sufficient to offset energy deficit.<br>American journal of physiology. Endocrinology and metabolism Jan 2006;290(1):E149-E153, 2006 Jan	2006	We evaluated brain uptake of endogenously produced lactate, alanine, and leucine
Lucidi P.; Rossetti P.; Porcellati F.; Pampanelli S.; Candeloro P.; Andreoli AM.; Perriello G.; Bolli GB.; Fanelli CG.<br>Mechanisms of insulin resistance after insulin-induced hypoglycemia in humans: the role of lipolysis.<br>Diabetes Jun 2010;59(6):1349-57, 2010 Jun	2010	To investigate the mechanisms of insulin resistance after insulin-induced hypoglycemia in humans
Lucidi P.; Murdolo G.; Di Loreto C.; De Cicco A.; Parlanti N.; Fanelli C.; Santeusanio F.; Bolli GB.; De Feo P.<br>Ghrelin is not necessary for adequate hormonal counterregulation of insulin-induced hypoglycemia.<br>Diabetes Oct 2002;51(10):2911-4, 2002 Oct	2002	To investigate if ghrelin is necessary for adequate hormonal counterregulation of insulin-induced hypoglycemia
Lunetta M.; Di Mauro M.; Le Moli R.; Nicoletti F.<br>Effect of octreotide on growth hormone, IGF-I, IGFBP-3, glucagon, cortisol and epinephrine response to insulin-induced hypoglycaemia in insulin-dependent diabetic patients.<br>Diabetes & metabolism Dec 1997;23(6):524-71997 Dec	1997	To evalute the effect of octreotide on growth hormone, IGF-I, IGFBP-3, glucagon, cortisol and epinephrine response to insulin-induced hypoglycaemia
Ly TT.; Jones TW.; Griffiths A.; Dart J.; Davis EA.; Stick S.; Wilson A.<br>Hypoglycemia does not change the threshold for arousal from sleep in adolescents with type 1 diabetes.<br>Diabetes technology & therapeutics Feb 2012;14(2):101-4, 2012 Feb	2012	To test the effect of hypoglycaemia on arousal from sleep
Ly TT.; Hewitt J.; Davey RJ.; Lim EM.; Davis EA.; Jones TW.<br>Improving epinephrine responses in hypoglycemia unawareness with real-time continuous glucose monitoring in adolescents with type 1 diabetes.<br>Diabetes care Jan 2011;34(1):50-2, 2011 Jan	2011	To determine whether real-time continuous glucose monitoring (CGM) with preset alarms at specific glucose levels would prove a useful tool to achieve avoidance of hypoglycemia

MacLeod KM.; Gold AE.; Ebmeier KP.; Hepburn DA.; Deary IJ.; Goodwin GM.; Frier BM.<br>The effects of acute hypoglycemia on relative cerebral blood flow distribution in patients with type I (insulin-dependent) diabetes and impaired hypoglycemia awareness.<br>Metabolism: clinical and experimental Aug 1996;45(8):974-80<br>1996 Aug	1996	The effects of acute hypoglycemia on relative cerebral blood flow distribution
Maggs DG.; Macdonald IA.; Tattersall RB.<br>Thermoregulatory responses to hyperinsulinaemic hypoglycaemia and euglycaemia in IDDM.<br>Diabetologia Jul 1994;37(7):689-96, 1994 Jul	1994	To characterize in more detail the thermoregulatory changes in human subjects during hypoglycaemia
Maggs DG.; Scott AR.; MacDonald IA.<br>Thermoregulatory responses to hyperinsulinemic hypoglycemia and euglycemia in humans.<br>The American journal of physiology Nov 1994;267(5 Pt 2):R1266-72<br>1994 Nov	1994	Examined the thermoregulatory responses to sustained hyperinsulinaemic hypoglycaemia and euglycaemia
Maggs DG.; Jacob R.; Rife F.; Caprio S.; Tamborlane WV.; Sherwin RS.<br>Counterregulation in peripheral tissues: effect of systemic hypoglycemia on levels of substrates and catecholamines in human skeletal muscle and adipose tissue.<br>Diabetes Jan 1997;46(1):70-6, 1997 Jan	1997	To distinguish the effects of hyperinsulinemia and hypoglycemia on glucose, gluconeogenic substrate, and catecholamine levels in adipose and muscle extracellular fluid
Mangia S.; Tesfaye N.; De Martino F.; Kumar AF.; Kollasch P.; Moheet AA.; Eberly LE.; Seaquist ER.<br>Hypoglycemia-induced increases in thalamic cerebral blood flow are blunted in subjects with type 1 diabetes and hypoglycemia unawareness.<br>Journal of cerebral blood flow and metabolism : official journal of the International Society of Cerebral Blood Flow and Metabolism Nov 2012;32(11):2084-90, 2012 Nov.	2012	The effect of hypoglycaemia on the thalamic cerebral blood flow
Maran A.; Lomas J.; Macdonald IA.; Amiel SA.<br>Lack of preservation of higher brain function during hypoglycaemia in patients with intensively-treated IDDM.<br>Diabetologia Dec 1995;38(12):1412-8, 1995 Dec	1995	To investigate the effect of diabetes control on higher brain function during acute hypoglycaemia
Maran A.; Crepaldi C.; Trupiani S.; Lucca T.; Jori E.; Macdonald IA.; Tiengo A.; Avogaro A.; Del Prato S.<br>Brain function rescue effect of lactate following hypoglycaemia is not an adaptation process in both normal and type I diabetic subjects.<br>Diabetologia Jun 2000;43(6):733-41, 2000 Jun	2000	To test if there is an lactate adaption during hypoglycaemia
Maran A.; Crepaldi C.; Del Piccolo F.; Macdonald I.; Zarantonello L.; Avogaro A.; Amodio P.<br>Cognitive, neurophysiologic and metabolic sequelae of previous hypoglycemic coma revealed by hyperinsulinemic-hypoglycemic clamp in type 1 diabetic patients.<br>Metabolic brain disease 10 2017;32(5):1543-1551, 2017 10	2017	To examine the relationship between electroencephalographi c (EEG) activity and hypoglycemia unawareness
Marques JL.; George E.; Peacey SR.; Harris ND.; Macdonald IA.; Cochrane T.; Heller SR.<br>Altered ventricular repolarization during hypoglycaemia in patients with diabetes.<br>Diabetic medicine : a journal of the British Diabetic Association Aug 1997;14(8):648-54, 1997 Aug	1997	The effects of hypoglycaemia on the electrocardiogram
Matyka K.; Evans M.; Lomas J.; Cranston I.; Macdonald I.; Amiel SA.	1997	To investigate the effect of normal aging

Altered hierarchy of protective responses against severe hypoglycemia in normal aging in healthy men.<br>Diabetes care Feb 1997;20(2):135-41, 1997 Feb		on the protective responses against hypoglycemia
M'bemba J.; Cynober L.; de Bandt P.; Taverna M.; Chevalier A.; Bardin C.; Slama G.; Selam JL.<br>Effects of dipeptide administration on hypoglycaemic counterregulation in type 1 diabetes.<br>Diabetes & metabolism Sep 2003;29(4 Pt 1):412-7, 2003 Sep	2003	To investigate if a dipeptide made of glutamine and alanine is able to contribute to the recovery from insulin-induced hypoglycaemia in type 1 diabetes
McAulay V.; Deary IJ.; Ferguson SC.; Frier BM.<br>Acute hypoglycemia in humans causes attentional dysfunction while nonverbal intelligence is preserved.<br>Diabetes care Oct 2001;24(10):1745-50, 2001 Oct	2001	Examines the effects of acute insulin-induced hypoglycemia on attention and intelligence in nondiabetic humans
McAulay V.; Deary IJ.; Sommerfield AJ.; Frier BM.<br>Attentional functioning is impaired during acute hypoglycaemia in people with Type 1 diabetes.<br>Diabetic medicine : a journal of the British Diabetic Association Jan 2006;23(1):26-31, 2006 Jan.	2006	To examine the effects of acute insulin-induced hypoglycaemia on different aspects of attention and on general non-verbal reasoning
McCrimmon RJ.; Frier BM.; Deary IJ.<br>Appraisal of mood and personality during hypoglycaemia in human subjects.<br>Physiology & behavior Aug 1999;67(1):27-33, 1999 Aug	1999	To examine the effect of a manipulation in mood-state on appraisal
McCrimmon RJ.; Ewing FM.; Frier BM.; Deary IJ.<br>Anger state during acute insulin-induced hypoglycaemia.<br>Physiology & behavior Aug 1999;67(1):35-9, 1999 Aug	1999	To examine the effects of insulin-induced hypoglycaemia on anger state
McGregor VP.; Banarer S.; Cryer PE.<br>Elevated endogenous cortisol reduces autonomic neuroendocrine and symptom responses to subsequent hypoglycemia.<br>American journal of physiology. Endocrinology and metabolism Apr 2002;282(4):E770-7, 2002 Apr	2002	The effect of elevated endogenous cortisol on autonomic neuroendocrine and symptom responses to subsequent hypoglycemia.
McGregor VP.; Greiwe JS.; Banarer S.; Cryer PE.<br>Limited impact of vigorous exercise on defenses against hypoglycemia: relevance to hypoglycemia-associated autonomic failure.<br>Diabetes May 2002;51(5):1485-92, 2002 May	2002	The effect of cortisol response to hypoglycemia on neurogenic symptom response to subsequent hypoglycemia
Mellman MJ.; Davis MR.; Shamoon H.<br>Effect of physiological hyperinsulinemia on counterregulatory hormone responses during hypoglycemia in humans.<br>The Journal of clinical endocrinology and metabolism Nov 1992;75(5):1293-7, 1992 Nov	1992	We evaluated the effect of hyperinsulinemia on counterregulatory hormone responses during hypoglycemia
Mellman MJ.; Davis MR.; Brisman M.; Shamoon H.<br>Effect of antecedent hypoglycemia on cognitive function and on glycemic thresholds for counterregulatory hormone secretion in healthy humans.<br>Diabetes care Mar 1994;17(3):183-8, 1994 Mar	1994	To determine whether reduced hormonal, symptomatic, and/or cognitive responses to hypoglycemia are caused by an increase in the plasma glucose concentration
Meneilly GS.; Cheung E.; Tuokko H.	1994	To investigate counterregulatory

Counterregulatory hormone responses to hypoglycemia in the elderly patient with diabetes.<br>Diabetes Mar 1994;43(3):403-10, 1994 Mar		hormone responses to hypoglycemia in elderly patients
Meneilly GS.; Cheung E.; Tuokko H.<br>Altered responses to hypoglycemia of healthy elderly people.<br>The Journal of clinical endocrinology and metabolism Jun 1994;78(6):1341-8, 1994 Jun	1994	To assess whether alterations in counterregulatory hormone release, decreased awareness of warning symptoms or alterations in psychomotor performance might increase the susceptibility of the elderly to hypoglycemia
Merl V.; Kern W.; Peters A.; Oltmanns KM.; Gais S.; Born J.; Fehm HL.; Schultes B.<br>Differences between nighttime and daytime hypoglycemia counterregulation in healthy humans.<br>Metabolism: clinical and experimental Jul 2004;53(7):894-8, 2004 Jul	2004	To determine differences between nighttime and daytime hypoglycemia counterresgulation
Meyer C.; Grossmann R.; Mitrakou A.; Mahler R.; Veneman T.; Gerich J.; Bretzel RG.<br>Effects of autonomic neuropathy on counterregulation and awareness of hypoglycemia in type 1 diabetic patients.<br>Diabetes care Nov 1998;21(11):1960-6, 1998 Nov	1998	The effect of autonomic neuropathy on counterregulation and awareness of hypoglycemia
Meyer C.; Hering BJ.; Grossmann R.; Brandhorst H.; Brandhorst D.; Gerich J.; Federlin K.; Bretzel RG.<br>Improved glucose counterregulation and autonomic symptoms after intraportal islet transplants alone in patients with long-standing type I diabetes mellitus.<br>Transplantation Jul 1998;66(2):233-40, 1998 Jul	1998	To asses if successful intraportal islet transplantation affect hormonal counterregulatory responses and symptoms
Meyer C.; Dostou JM.; Gerich JE.<br>Role of the human kidney in glucose counterregulation.<br>Diabetes May 1999;48(5):943-8 1999 May	1999	Whether the kidney is also involved in human glucose counterregulation
Meyer C.; Saar P.; Soydan N.; Eckhard M.; Bretzel RG.; Gerich J.; Linn T.<br>A potential important role of skeletal muscle in human counterregulation of hypoglycemia.<br>The Journal of clinical endocrinology and metabolism Nov 2005;90(11):6244-50, 2005 Nov	2005	The contribution of skeletal muscle to glucose counterregulation in humans.
Mikeladze M.; Hedrington MS.; Joy N.; Tate DB.; Younk LM.; Davis I.; Davis SN.<br>Acute Effects of Oral Dehydroepiandrosterone on Counterregulatory Responses During Repeated Hypoglycemia in Healthy Humans.<br>Diabetes 10 2016;65(10):3161-70, 2016 10	2016	Acute Effects of Oral Dehydroepiandrosterone on Counterregulatory Responses
Mitrakou A.; Ryan C.; Veneman T.; Mokan M.; Jenssen T.; Kiss I.; Durrant J.; Cryer P.; Gerich J.<br>Hierarchy of glycemic thresholds for counterregulatory hormone secretion, symptoms, and cerebral dysfunction.<br>The American journal of physiology Jan 1991;260(1 Pt 1):E67-74, 1991 Jan	1991	To investigate hierarchy of glycemic thresholds for counterregulatory hormone secretion
Milman S.; Leu J.; Shamoon H.; Vele S.; Gabriely I.<br>Opioid receptor blockade prevents exercise-associated autonomic failure in humans.<br>Diabetes Jun 2012;61(6):1609-15, 2012 Jun	2012	The effect of opioid receptor blockade on autonomic failure during exercise
Moberg E.; Kollind M.; Ostenson CG.; Lins PE.; Adamson U.	1996	To evaluate the effect of the alfa2-adrenoreceptor antagonist idazoxan on

Acute effects of the alpha 2-adrenoreceptor antagonist idazoxan on hormonal responses and symptoms of hypoglycaemia in patients with type 1 diabetes mellitus.<br>Diabetes & metabolism Feb 1996;22(1):31-6, 1996 Feb		hormonal responses and symptoms of hypoglycaemia
Moberg E.; Hagström-Toft E.; Bolinder J.<br>Detection of hypoglycaemia by microdialysis measurements of glucose in subcutaneous adipose tissue.<br>Hormone and metabolic research = Hormon- und Stoffwechselforschung = Hormones et metabolisme Sep 1997;29(9):440-3, 1997 Sep	1997	The use of various fractional sampling periods for the detection of hypoglycaernia by microdialysis of the adipose tissue
Moberg E.; Hagström-Toft E.; Arner P.; Bolinder J.<br>Protracted glucose fall in subcutaneous adipose tissue and skeletal muscle compared with blood during insulin-induced hypoglycaemia.<br>Diabetologia Nov 1997;40(11):1320-6, 1997 Nov	1997	The effect of glucose concentrations in subcutaneous adipose tissue and skeletal muscle
Mokan M.; Mitrakou A.; Veneman T.; Ryan C.; Korytkowski M.; Cryer P.; Gerich J.<br>Hypoglycemia unawareness in IDDM.<br>Diabetes care Dec 1994;17(12):1397-403, 1994 Dec	1994	To assess the characteristics of patients with hypoglycemia unawareness
Moriarty KT.; Simpson EJ.; Brown NS.; Macdonald IA.; Tattersall RB.<br>Effect of acute mild hypoglycaemia on counterregulatory responses to moderate hypoglycaemia induced immediately afterwards in healthy men.<br>Clinical science (London, England : 1979) Nov 1993;85(5):537-42, 1993 Nov	1993	To determine whether a 1 h period of mild hypoglycaemia affected the response to an episode of moderate hypoglycaemia immediately afterwards
Moriarty KT.; Simpson EJ.; Mullinger RN.; MacDonald IA.; Tattersall RB.<br>Antecedent insulin level and pattern of induction of acute hypoglycaemia do not affect subsequent counterregulatory responses in healthy subjects.<br>Clinical science (London, England : 1979) Nov 1993;85(5):543-8, 1993 Nov	1993	To determine whether the duration and pattern of prior insulin exposure modulate the symptomatic and counterregulatory responses
Morrow L.; Hompesch M.; Tideman AM.; Matson J.; Dunne N.; Pardo S.; Parkes JL.; Schachner HC.; Simmons DA.<br>Evaluation of a novel continuous glucose measurement device in patients with diabetes mellitus across the glycemic range.<br>Journal of diabetes science and technology Jul 2011;5(4):853-9, 2011 Jul	2011	Assessed the performance of an electrochemical continuous glucose monitoring (CGM) system
Monsod TP.; Flanagan DE.; Rife F.; Saenz R.; Caprio S.; Sherwin RS.; Tamborlane WV.<br>Do sensor glucose levels accurately predict plasma glucose concentrations during hypoglycemia and hyperinsulinemia?<br>Diabetes care May 2002;25(5):889-93, 2002 May	2002	To examine whether the relationship between plasma and interstitial fluid glucose is altered by changes in plasma glucose and insulin levels
Mumme L.; Breuer TGK.; Rohrer S.; Schenker N.; Menge BA.; Holst JJ.; Nauck MA.; Meier JJ.<br>Defects in α-Cell Function in Patients With Diabetes Due to Chronic Pancreatitis Compared With Patients With Type 2 Diabetes and Healthy Individuals.<br>Diabetes care 10 2017;40(10):1314-1322, 2017 1	2017	Examined the alterations in the glucagon response to hypoglycemia and to oral glucose administration
Musen G.; Simonson DC.; Bolo NR.; Driscoll A.; Weinger K.; Raji A.; Théberge J.; Renshaw PF.; Jacobson AM.<br>Regional brain activation during hypoglycemia in type 1 diabetes.<br>The Journal of clinical endocrinology and metabolism Apr 2008;93(4):1450-7, 2008 Apr	2008	To determine the blood glucose level at which the hypothalamus and other brain regions are activated in response to hypoglycemia

Møller J.; Laker MF.; Gillespie SM.; Ovesen PG.; Abildgaard N.; Tian R.; Jørgensen JO.; Møller N.<br>Lack of effects of hypoglycemia on glucose absorption in healthy men.<br>Diabetes care Oct 1992;15(10):1264-6, 1992 Oct	1992	To assess the effects of hypoglycemia on glucose absorption by examining the systemic appearance of 3-OMG
Naik S.; Belfort-DeAguiar R.; Sejling AS.; Szepietowska B.; Sherwin RS.<br>Evaluation of the counter-regulatory responses to hypoglycaemia in patients with type 1 diabetes during opiate receptor blockade with naltrexone.<br>Diabetes, obesity & metabolism 05 2017;19(5):615-621 2017 05	2017	To investigate if the oral formulation of the long acting opiate antagonist, naltrexone, could have a similar effect, and thus might be useful therapeutically
Nauck MA.; Heimesaat MM.; Behle K.; Holst JJ.; Nauck MS.; Ritzel R.; Hüfner M.; Schmiegel WH.<br>Effects of glucagon-like peptide 1 on counterregulatory hormone responses, cognitive functions, and insulin secretion during hyperinsulinemic, stepped hypoglycemic clamp experiments in healthy volunteers.<br>The Journal of clinical endocrinology and metabolism Mar 2002;87(3):1239-46 2002 Mar	2002	To investigate if GLP-1 suppresses glucagon secretion, which could lead to disturbances of hypoglycemia
Nermoen I.; Jorde R.; Sager G.; Sundsfjord J.; Birkeland K.<br>Effects of exercise on hypoglycaemic responses in insulin-dependent diabetes mellitus.<br>Diabetes & metabolism Apr 1998;24(2):131-6, 1998 Apr	1998	To determine whether moderate exercise influences hypoglycaemic responses in IDDM
Norjavaara E.; Ericsson H.; Sjöberg F.; Leonsson-Zachrisson M.; Sjöstrand M.; Morrow LA.; Hompesch M.<br>Glucokinase activators AZD6370 and AZD1656 do not affect the central counterregulatory response to hypoglycemia in healthy males.<br>The Journal of clinical endocrinology and metabolism Sep 2012;97(9):3319-25 2012 Sep	2012	Two separate studies assessed the counterregulatory hormone responses to hypoglycemia induced by the GKAs
Novodvorsky P.; Bernjak A.; Robinson EJ.; Iqbal A.; Macdonald IA.; Jacques RM.; Marques JLB.; Sheridan PJ.; Heller SR.<br>Salbutamol-induced electrophysiological changes show no correlation with electrophysiological changes during hyperinsulinaemic-hypoglycaemic clamp in young people with Type 1 diabetes.<br>Diabetic medicine : a journal of the British Diabetic Association Apr 2018;(): 2018 Apr	2018	Investigation of salbutamol-induced ECG changes compared with ECG changes during hypoglycaemia.
Nyholm B.; Møller N.; Gravholt CH.; Orskov L.; Mengel A.; Bryan G.; Moyses C.; Alberti KG.; Schmitz O.<br>Acute effects of the human amylin analog AC137 on basal and insulin-stimulated euglycemic and hypoglycemic fuel metabolism in patients with insulin-dependent diabetes mellitus.<br>The Journal of clinical endocrinology and metabolism Mar 1996;81(3):1083-9, 1996 Mar	1996	To assess the acute effect of iv infused AC137
Orskov L.; Bak JF.; Abildgård .; Schmitz O.; Andreasen F.; Richter EA.; Skjaerbaek C.; Møller N.<br>Inhibition of muscle glycogen synthase activity and non-oxidative glucose disposal during hypoglycaemia in normal man.<br>Diabetologia Feb 1996;39(2):226-34, 1996 Feb	1996	To evaluate the role of muscle glycogen synthase activity in the reduction of glucose uptake during hypoglycaemia
Orskov L.; Schmitz O.; Bak JF.; Lund S.; Kaal A.; Nyholm B.; Møller N.<br>Skeletal muscle glucose uptake, glycogen synthase activity and GLUT 4 content during hypoglycaemia in type 1 diabetic subjects.	2001	to examine the efficacy of skeletal muscle glucose uptake, glycogen synthase and GLUT4
Ovalle F.; Fanelli CG.; Paramore DS.; Hershey T.; Craft S.; Cryer PE.	1998	To assess the impact of hypoglycemia-associated autonomic

Brief twice-weekly episodes of hypoglycemia reduce detection of clinical hypoglycemia in type 1 diabetes mellitus.<br>Diabetes Sep 1998;47(9):1472-9, 1998 Sep		failure on the daily lives of people with DM1
Oltmanns KM.; Deininger E.; Wellhoener P.; Schultes B.; Kern W.; Marx E.; Dominiak P.; Born J.; Fehm HL.; Peters A.<br>Influence of captopril on symptomatic and hormonal responses to hypoglycaemia in humans.<br>British journal of clinical pharmacology Apr 2003;55(4):347-53, 2003 Apr	2003	The effect of captopril on hypoglycaemia
Oltmanns KM.; Gehring H.; Rudolf S.; Schultes B.; Rook S.; Schweiger U.; Born J.; Fehm HL.; Peters A.<br>Hypoxia causes glucose intolerance in humans.<br>American journal of respiratory and critical care medicine Jun 2004;169(11):1231-7, 2004 Jun	2004	To investigate if hypoxia affects the glucose intolerance
Oltmanns KM.; Fruehwald-Schultes B.; Kern W.; Born J.; Fehm HL.; Peters A.<br>Hypoglycemia, but not insulin, acutely decreases LH and T secretion in men.<br>The Journal of clinical endocrinology and metabolism Oct 2001;86(10):4913-9, 2001 Oct	2001	Whether insulin or changes in blood glucose levels influence pituitary gonadotropin secretion or testicular steroidogenesis in healthy men
Oz G.; Kumar A.; Rao JP.; Kodl CT.; Chow L.; Eberly LE.; Seaquist ER.<br>Human brain glycogen metabolism during and after hypoglycemia.<br>Diabetes Sep 2009;58(9):1978-85, 2009 Sep	2009	We tested if human brain glycogen is mobilized during hypoglycemia
Page KA.; Williamson A.; Yu N.; McNay EC.; Dzuira J.; McCrimmon RJ.; Sherwin RS.<br>Medium-chain fatty acids improve cognitive function in intensively treated type 1 diabetic patients and support in vitro synaptic transmission during acute hypoglycemia.<br>Diabetes May 2009;58(5):1237-44, 2009 May	2009	We examined whether ingestion of medium-chain triglycerides could improve cognition during hypoglycemia
Page KA.; Seo D.; Belfort-DeAguiar R.; Lacadie C.; Dzuira J.; Naik S.; Amarnath S.; Constable RT.; Sherwin RS.; Sinha R.<br>Circulating glucose levels modulate neural control of desire for high-calorie foods in humans.<br>The Journal of clinical investigation Oct 2011;121(10):4161-9, 2011 Oct	2011	To investigate the hypothesis if circulating levels of glucose influence brain regions that regulate the motivation to consume high-calorie foods
Pais I.; Hubold C.; Hallschmid M.; Letterer S.; Oltmanns K.; Schultes B.; Born J.; Peters A.<br>Blocking NMDA receptor signaling does not decrease hormonal counterregulation to hypoglycemia in humans.<br>Psychoneuroendocrinology Sep 2008;33(8):1069-76 2008 Sep	2008	We examined the effect of the NMDA receptor antagonist memantine
Paramalingam N.; Fournier PA.; Davey RJ.; Roby HC.; Smith GJ.; Shetty VB.; Guelfi KJ.; Davis EA.; Jones TW.<br>A 10-second sprint does not blunt hormonal counter-regulation to subsequent hypoglycaemia.<br>Diabetic medicine : a journal of the British Diabetic Association 10 2017;34(10):1440-1446, 2017 10	2017	To investigate whether a 10-second sprint impairs the counter-regulatory response to subsequent hypoglycaemia
Paramore DS.; Fanelli CG.; Shah SD.; Cryer PE.<br>Forearm norepinephrine spillover during standing, hyperinsulinemia, and hypoglycemia.<br>The American journal of physiology Nov 1998;275(5 Pt 1):E872-81<br>1998 Nov	1998	To distinguish sympathetic neural from adrenomedullary activation during prolonged standing, hyperinsulinemic euglycemia, and hyperinsulinemic hypoglycemia
Paramore DS.; Fanelli CG.; Shah SD.; Cryer PE.	1999	To assess if hypoglycemia per se

Hypoglycemia per se stimulates sympathetic neural as well as adrenomedullary activity, but, unlike the adrenomedullary response, the forearm sympathetic neural response is not reduced after recent hypoglycemia.<br>Diabetes Jul 1999;48(7):1429-36, 1999 Jul		stimulates sympathetic neural as well as adrenomedullary activity
Passias TC.; Meneilly GS.; Mekjavić IB.<br>Effect of hypoglycemia on thermoregulatory responses.<br>Journal of applied physiology (Bethesda, Md. : 1985) Mar 1996;80(3):1021-32, 1996 Mar	1996	The effects of hypoglycaemia on automatic thermoregulatory responses
Paty BW.; Ryan EA.; Shapiro AM.; Lakey JR.; Robertson RP.<br>Intrahepatic islet transplantation in type 1 diabetic patients does not restore hypoglycemic hormonal counterregulation or symptom recognition after insulin independence.<br>Diabetes Dec 2002;51(12):3428-34, 2002 Dec	2002	To determine if hypoglycemic counterregulation is restored by islet transplantation
Peacey SR.; George E.; Rostami-Hodjegan A.; Bedford C.; Harris N.; Hardisty CA.; Tucker GT.; Macdonald IA.; Heller SR.<br>Similar physiological and symptomatic responses to sulphonylurea and insulin induced hypoglycaemia in normal subjects.<br>Diabetic medicine : a journal of the British Diabetic Association Jul 1996;13(7):634-41, 1996 Jul	1996	We compared the physiological and symptomatic responses to insulin and tolbutamide
Peacey SR.; Rostami-Hodjegan A.; George E.; Tucker GT.; Heller SR.<br>The use of tolbutamide-induced hypoglycemia to examine the intraislet role of insulin in mediating glucagon release in normal humans.<br>The Journal of clinical endocrinology and metabolism May 1997;82(5):1458-61,1997 May	1997	To investigate the intraislet role of insulin by using tolbutamide-induced hypoglycaemia
Pieber TR.; Deller S.; Korsatko S.; Jensen L.; Christiansen E.; Madsen J.; Heller SR.<br>Counter-regulatory hormone responses to hypoglycaemia in people with type 1 diabetes after 4 weeks of treatment with liraglutide adjunct to insulin: a randomized, placebo-controlled, double-blind, crossover trial.<br>Diabetes, obesity & metabolism Aug 2015;17(8):742-5, 2015 Aug	2015	To investigate the effect of glucagon-like peptide 1 receptor agonist liraglutide on the counter-regulatory hormone response
Pitsillides AN.; Anderson SM.; Kovatchev B.<br>Hypoglycemia risk and glucose variability indices derived from routine self-monitoring of blood glucose are related to laboratory measures of insulin sensitivity and epinephrine counterregulation.<br>Diabetes technology & therapeutics Jan 2011;13(1):11-7, 2011 Jan	2011	Investigate possible relationships between outpatient measures of glucose variability and risk for hypoglycemia
Plummer MP.; Jones KL.; Annink CE.; Cousins CE.; Meier JJ.; Chapman MJ.; Horowitz M.; Deane AM.<br>Glucagon-like peptide 1 attenuates the acceleration of gastric emptying induced by hypoglycemia in healthy subjects.<br>Diabetes care Jun 2014;37(6):1509-15, 2014 Jun	2014	To determine whether GLP-1 attenuates the acceleration of gastric emptying induced by hypoglycemia
Poulsen PL.; Orskov L.; Grøfte T.; Møller J.; Holst JJ.; Schmitz O.; Møller N.<br>Effects of oral glucose on systemic glucose metabolism during hyperinsulinemic hypoglycemia in normal man.<br>Metabolism: clinical and experimental Dec 2000;49(12):1598-603<br>2000 Dec	2000	To define the systemic impact and time course of effects following oral glucose during hypoglycemia
Powers WJ.; Boyle PJ.; Hirsch IB.; Cryer PE.<br>Unaltered cerebral blood flow during hypoglycemic activation of the sympathochromaffin system in humans.<br>The American journal of physiology Oct 1993;265(4 Pt 2):R883-7<br>1993 Oct	1993	To determine if increases in plasma epinephrine and norepinephrine caused by hypoglycemia are associated with increments in cerebral blood flow
Qvisth V.; Hagström-Toft E.; Enoksson S.; Moberg E.; Arner P.; Bolinder J.<br>Human skeletal muscle lipolysis is more responsive to epinephrine than to norepinephrine stimulation in vivo.	2006	To investigate the effect of endogenous catecholamines on

The Journal of clinical endocrinology and metabolism Feb 2006;91(2):665-70, 2006 Feb		TG lipolysis in human SM in vivo
Qvisth V.; Hagström-Toft E.; Enoksson S.; Bolinder J.<br>Catecholamine regulation of local lactate production in vivo in skeletal muscle and adipose tissue: role of -adrenoreceptor subtypes.<br>The Journal of clinical endocrinology and metabolism Jan 2008;93(1):240-6, 2008 Jan	2008	To investigate the catecholamine-mediated regulation of lactate production and blood flow in SM and AT
Raju B.; McGregor VP.; Cryer PE.<br>Cortisol elevations comparable to those that occur during hypoglycemia do not cause hypoglycemia-associated autonomic failure.<br>Diabetes Aug 2003;52(8):2083-9, 2003 Aug	2003	To assess the suggestion that it is the cortisol response to antecedent hypoglycem ia that mediates HAAF
Ramanathan R.; Cryer PE.<br>Adrenergic mediation of hypoglycemia-associated autonomic failure.<br>Diabetes Feb 2011;60(2):602-6, 2011 Feb	2011	The effect of adrenergic mediation of hypoglycemia-associated autonomic failure
Rana O.; Byrne CD.; Kerr D.; Coppini DV.; Zouwail S.; Senior R.; Begley J.; Walker JJ.; Greaves K.<br>Acute hypoglycemia decreases myocardial blood flow reserve in patients with type 1 diabetes mellitus and in healthy humans.<br>Circulation Oct 2011;124(14):1548-56, 2011 Oct	2011	To assess the myocardial blood flow reserve is during hypoglycemia
Rao AD.; Bonyhay I.; Dankwa J.; Baimas-George M.; Kneen L.; Ballatori S.; Freeman R.; Adler GK.<br>Baroreflex Sensitivity Impairment During Hypoglycemia: Implications for Cardiovascular Control.<br>Diabetes Jan 2016;65(1):209-15 2016 Jan	2016	To determine the acute effects of hypoglycemia on cardiovascular autonomic control
Rasmussen BM.; Orskov L.; Schmitz O.; Hermansen K.<br>Alcohol and glucose counterregulation during acute insulin-induced hypoglycemia in type 2 diabetic subjects.<br>Metabolism: clinical and experimental Apr 2001;50(4):451-7, 2001 Apr	2001	To investigate the influence of alcohol on glucose counterregulation and recovery during acute insulin-induced hypoglycemia
Rattarasarn C.; Dagogo-Jack S.; Zachwieja JJ.; Cryer PE.<br>Hypoglycemia-induced autonomic failure in IDDM is specific for stimulus of hypoglycemia and is not attributable to prior autonomic activation.<br>Diabetes Jun 1994;43(6):809-18, 1994 Jun	1994	To assess that recent antecedent hypoglycemia causes reduced autonomic responses to subsequent hypoglycemia
Ratter JM.; Rooijackers HM.; Tack CJ.; Hijmans AG.; Netea MG.; de Galan BE.; Stienstra R.<br>Proinflammatory Effects of Hypoglycemia in Humans With or Without Diabetes.<br>Diabetes 04 2017;66(4):1052-1061, 2017 04	2017	To test whether the composition and inflammatory function of immune cells adapt to a more proinflammatory state after hypoglycemia
Rickels MR.; Schutta MH.; Mueller R.; Markmann JF.; Barker CF.; Naji A.; Teff KL.<br>Islet cell hormonal responses to hypoglycemia after human islet transplantation for type 1 diabetes.<br>Diabetes Nov 2005;54(11):3205-11, 2005 Nov	2005	We studied the glucagon response after human islet transplantation for type 1 diabetes
Rickels MR.; Schutta MH.; Mueller R.; Kapoor S.; Markmann JF.; Naji A.; Teff KL.<br>Glycemic thresholds for activation of counterregulatory hormone and symptom responses in islet transplant recipients.<br>The Journal of clinical endocrinology and metabolism Mar 2007;92(3):873-9, 2007 Mar	2007	To determine whether the avoidance of hypoglycemia achiev ed through islet transplantation results in improved glycemic thresholds for

		counterregulatory resp onses
Rickels MR.; Fuller C.; Dalton-Bakes C.; Markmann E.; Palanjian M.; Cullison K.; Tiao J.; Kapoor S.; Liu C.; Naji A.; Teff KL.<br>Restoration of Glucose Counterregulation by Islet Transplantation in Long-standing Type 1 Diabetes.<br>Diabetes May 2015;64(5):1713-8, 2015 May	2015	Whether intrahepatic islets respond appropriately to hypoglycemia after transplantation has not been fully studied
Robinson AM.; Parkin HM.; Macdonald IA.; Tattersall RB.<br>Physiological response to postural change during mild hypoglycaemia in patients with IDDM.<br>Diabetologia Dec 1994;37(12):1241-50, 1994 Dec	1994	The effect of posture and duration of hypoglycaemia on symptoms and physiological responses
Robinson RT.; Harris ND.; Ireland RH.; Lee S.; Newman C.; Heller SR.<br>Mechanisms of abnormal cardiac repolarization during insulin-induced hypoglycemia.<br>Diabetes Jun 2003;52(6):1469-74, 2003 Jun	2003	We measured cardiac repol arization (QT interval [QTc] and QT dispersion [QTd]) during experimental hypoglyce mia with and without beta-blockade and potassium infusion to establish possible mechanisms
Rooijackers HM.; Wiegers EC.; van der Graaf M.; Thijssen DH.; Kessels RPC.; Tack CJ.; de Galan BE.<br>A Single Bout of High-Intensity Interval Training Reduces Awareness of Subsequent Hypoglycemia in Patients With Type 1 Diabetes.<br>Diabetes 07 2017;66(7):1990-1998 2017 07	2017	The effect of HIIT on awareness of hypoglycemia and hypoglycemia-induced cognitive dysfunction
Rosenn BM.; Miodovnik M.; Khoury JC.; Siddiqi TA.<br>Counterregulatory hormonal responses to hypoglycemia during pregnancy.<br>Obstetrics and gynecology Apr 1996;87(4):568-74 1996 Apr	1996	To evaluate the counterregulatory responses to insulin-induced hypoglycemia in healthy women and in women with insulin-dependent diabetes during pregnancy and in the nonpregnant state
Rossetti P.; Porcellati F.; Busciantella Ricci N.; Candeloro P.; Cioli P.; Nair KS.; Santeusanio F.; Bolli GB.; Fanelli CG.<br>Effect of oral amino acids on counterregulatory responses and cognitive function during insulin-induced hypoglycemia in nondiabetic and type 1 diabetic people.<br>Diabetes Jul 2008;57(7):1905-17, 2008 Jul	2008	To assess the responses to hypoglycemia in nondiabetic and type 1 diabetic subjects after ingestion of an amino acid mixture
Russell RR.; Chyun D.; Song S.; Sherwin RS.; Tamborlane WV.; Lee FA.; Pfeifer MA.; Rife F.; Wackers FJ.; Young LH.<br>Cardiac responses to insulin-induced hypoglycemia in nondiabetic and intensively treated type 1 diabetic patients.<br>American journal of physiology. Endocrinology and metabolism Nov 2001;281(5):E1029-36, 2001 Nov	2001	To investigate the cardiac response during hypoglycaemia
Russo A.; Stevens JE.; Chen R.; Gentilcore D.; Burnet R.; Horowitz M.; Jones KL.<br>Insulin-induced hypoglycemia accelerates gastric emptying of solids and liquids in long-standing type 1 diabetes.<br>The Journal of clinical endocrinology and metabolism Aug 2005;90(8):4489-95, 2005 Aug	2005	The effect of insulin-induced hypoglycemia on gastric emptying of solids and liquids in long-standing type 1 diabetes.
Ryan CM.; Atchison J.; Puczynski S.; Puczynski M.; Arslanian S.; Becker D.<br>Mild hypoglycemia associated with deterioration of mental efficiency in children with insulin-dependent diabetes mellitus.	1990	To assess the effects of mild hypoglycemia on

The Journal of pediatrics Jul 1990;117(1 Pt 1):32-8, 1990 Jul		cognitive functioning in diabetic children
Ryan CM.; Dulay D.; Suprasongsin C.; Becker DJ.<br>Detection of symptoms by adolescents and young adults with type 1 diabetes during experimental induction of mild hypoglycemia: role of hormonal and psychological variables.<br>Diabetes care May 2002;25(5):852-8, 2002 May	2002	To identify hormonal, psychological, and demographic predictors of symptom detection and accuracy of blood glucose
Sandoval DA.; Ertl AC.; Richardson MA.; Tate DB.; Davis SN.<br>Estrogen blunts neuroendocrine and metabolic responses to hypoglycemia.<br>Diabetes Jul 2003;52(7):1749-55, 2003 Jul	2003	To determine whether estrogen is a major in vivo mechanism responsible for the sexual dimorphism
Sandoval DA.; Guy DL.; Richardson MA.; Ertl AC.; Davis SN.<br>Acute, same-day effects of antecedent exercise on counterregulatory responses to subsequent hypoglycemia in type 1 diabetes mellitus.<br>American journal of physiology. Endocrinology and metabolism Jun 2006;290(6):E1331-8, 2006 Jun	2006	To determine the acute effects of morning exercise or moderate hypoglycemia on autonomic, neuroendocrine, and metabolic responses
Sandoval DA.; Galassetti P.; Tate D.; Neill A.; Davis SN.<br>Leptin responses to antecedent exercise and hypoglycemia in healthy and type 1 diabetes mellitus men and women.<br>Journal of diabetes and its complications ;17(6):301-6	2003	To test the hypothesis that clinically relevant repeated stress, such as hypoglycemia or exercise, could blunt leptin's response to subsequent stress
Schafer RJ.; Page KA.; Arora J.; Sherwin R.; Constable RT.<br>BOLD response to semantic and syntactic processing during hypoglycemia is load-dependent.<br>Brain and language Jan 2012;120(1):1-14, 2012 Jan	2012	This study investigates how syntactic and semantic load factors impact sentence comprehension and BOLD signal
Schächinger H.; Cox D.; Linder L.; Brody S.; Keller U.<br>Cognitive and psychomotor function in hypoglycemia: response error patterns and retest reliability.<br>Pharmacology, biochemistry, and behavior Jul 2003;75(4):915-20, 2003 Jul	2003	To test the cognitive and psychomotor function during hypoglycaemia
Sherr J.; Xing D.; Ruedy KJ.; Beck RW.; Kollman C.; Buckingham B.; White NH.; Fox L.; Tsalikian E.; Weinzimer S.; Arbelaez AM.; Tamborlane WV.; .<br>Lack of association between residual insulin production and glucagon response to hypoglycemia in youth with short duration of type 1 diabetes.<br>Diabetes care Jun 2013;36(6):1470-6, 2013 Jun	2013	To examine the loss of glucagon response to hypoglycemia and its relationship with residual β-cell function early in the course of type 1 diabetes (T1D) in youth
Schmid SM.; Jauch-Chara K.; Hallschmid M.; Schultes B.<br>Mild sleep restriction acutely reduces plasma glucagon levels in healthy men.<br>The Journal of clinical endocrinology and metabolism Dec 2009;94(12):5169-73, 2009 Dec	2009	To assess the effects of a single night of sleep restriction to 4.5 hon endocrine parameters of glucose metabolism
Schmid SM.; Hallschmid M.; Jauch-Chara K.; Bandorf N.; Born J.; Schultes B.<br>Sleep loss alters basal metabolic hormone secretion and modulates the dynamic counterregulatory response to hypoglycemia.<br>The Journal of clinical endocrinology and metabolism Aug 2007;92(8):3044-51, 2007 Aug	2007	The effect of sleep loss on basal metabolic hormone secretion and the dynamic counterregulatory response to hypoglycemia
Schvarcz E.; Palmér M.; Aman J.; Berne C.<br>Hypoglycemia increases the gastric emptying rate in healthy subjects.<br>Diabetes care May 1995;18(5):674-6 1995 May	1995	To compare the gastric emptying rate during hypoglycaemia to that during euglycaemia

Schmitz O.; Fisker S.; Orskov L.; Hove KY.; Nyholm B.; Møller N.<br>Effects of hyperinsulinaemia and hypoglycaemia on circulating leptin levels in healthy lean males.<br>Diabetes & metabolism Feb 1997;23(1):80-3, 1997 Feb	1997	To elucidate the role of short-term hypoinsulineamia and hypoglycaemia on circulating levels of leptin
Schmoller A.; Hass T.; Strugovshchikova O.; Melchert UH.; Scholand-Engler HG.; Peters A.; Schweiger U.; Hohagen F.; Oltmanns KM.<br>Evidence for a relationship between body mass and energy metabolism in the human brain.<br>Journal of cerebral blood flow and metabolism : official journal of the International Society of Cerebral Blood Flow and Metabolism Jul 2010;30(7):1403-10, 2010 Jul	2010	To examine whether there is a relationship between body mass and adenosine triphosphate (ATP) metabolism in the human brain
Schvarcz E.; Palmér M.; Aman J.; Berne C.<br>Atropine inhibits the increase in gastric emptying during hypoglycemia in humans.<br>Diabetes care Nov 1995;18(11):1463-7, 1995 Nov	1995	To study the effect of a cholinergic muscarinic blockade on the gastric emptying rate during insulin-induced hypoglycemia
Schvarcz E.; Palmér M.; Aman J.; Lindkvist B.; Beckman KW.<br>Hypoglycaemia increases the gastric emptying rate in patients with type 1 diabetes mellitus.<br>Diabetic medicine : a journal of the British Diabetic Association ;10(7):660-3	1993	To compare the gastric emptying rate during normoglycemia with that during insulin-induced hypoglycemia
Schmid SM.; Jauch-Chara K.; Hallschmid M.; Oltmanns KM.; Peters A.; Born J.; Schultes B.<br>Lactate overrides central nervous but not beta-cell glucose sensing in humans.<br>Metabolism: clinical and experimental Dec 2008;57(12):1733-9, 2008 Dec	2008	Examined whether lactate also interacts with pancreatic glucose-sensing mechanisms in vivo
Schopman JE.; Admiraal WM.; Soeters MR.; Ackermans MT.; Bisschop PL.; Frier BM.; Hoekstra JB.; Romijn JA.; Verberne HJ.; Holleman F.<br>(18)F-fluorodeoxyglucose uptake in brown adipose tissue during insulin-induced hypoglycemia and mild cold exposure in non-diabetic adults.<br>Metabolism: clinical and experimental Oct 2014;63(10):1280-6, 2014 Oct	2014	Examined the effect of hypoglycemia on uptake of the labeled glucose analogue 18 F-fluorodeoxyglucose in brown adipose tissue
Schouwenberg BJ.; Smits P.; Tack CJ.; de Galan BE.<br>The effect of antecedent hypoglycaemia on β_2-adrenergic sensitivity in healthy participants with the Arg16Gly polymorphism of the β_2-adrenergic receptor.<br>Diabetologia May 2011;54(5):1212-8, 2011 May	2011	We compared the effect of antecedent hypoglycaemia on β(2)-adrenergic receptor sensitivity between GlyGly participants and those with arginine 16
Schultes B.; Oltmanns KM.; Kern W.; Born J.; Fehm HL.; Peters A.<br>Acute and prolonged effects of insulin-induced hypoglycemia on the pituitary-thyroid axis in humans.<br>Metabolism: clinical and experimental Oct 2002;51(10):1370-4, 2002 Oct	2002	To study the effect of insulin-induced hypoglycaemia on the pituitary-thyroid axis
Schultes B.; Kern W.; Oltmanns K.; Peters A.; Gais S.; Fehm HL.; Born J.<br>Differential adaptation of neurocognitive brain functions to recurrent hypoglycemia in healthy men.<br>Psychoneuroendocrinology Feb 2005;30(2):149-61, 2005 Feb	2005	We compared the influence of recurrent hypoglycemia on counterregulatory hormones, subjective symptoms of hypoglycemia, short-term memory performance and performance on an auditory attention task
Schultes B.; Peters A.; Kern W.; Gais S.; Oltmanns KM.; Fehm HL.; Born J.<br>Processing of food stimuli is selectively enhanced during insulin-induced hypoglycemia in healthy men.	2005	To explore the neuroendocrine mechanisms mediating

Psychoneuroendocrinology Jun 2005;30(5):496-504, 2005 Jun		the acute effects of sleep deprivation on blood glucose regulation under basal and hypoglycemic conditions
Schwartz NS.; Clutter WE.; Shah SD.; Cryer PE.<br>Glycemic thresholds for activation of glucose counterregulatory systems are higher than the threshold for symptoms.<br>The Journal of clinical investigation Mar 1987;79(3):777-81, 1987 Mar	1987	To define glycemic thresholds for activation of glucose counterregulatory systems and for symptoms of hypoglycemia
Segel SA.; Fanelli CG.; Dence CS.; Markham J.; Videen TO.; Paramore DS.; Powers WJ.; Cryer PE.<br>Blood-to-brain glucose transport, cerebral glucose metabolism, and cerebral blood flow are not increased after hypoglycemia.<br>Diabetes Aug 2001;50(8):1911-7, 2001 Aug	2001	To assess if recent antecedent hypoglycemia increases blood-to-brain glucose transport
Segel SA.; Paramore DS.; Cryer PE.<br>Hypoglycemia-associated autonomic failure in advanced type 2 diabetes.<br>Diabetes Mar 2002;51(3):724-33, 2002 Mar	2002	The effect of hypoglycaemia on glucagon response in patients who are approaching the insulin-deficient end of the spectrum of type 2 diabetes
Sejling AS.; Kjær TW.; Pedersen-Bjergaard U.; Diemar SS.; Frandsen CS.; Hilsted L.; Faber J.; Holst JJ.; Tarnow L.; Nielsen MN.; Remvig LS.; Thorsteinsson B.; Juhl CB.<br>Hypoglycemia-associated changes in the electroencephalogram in patients with type 1 diabetes and normal hypoglycemia awareness or unawareness.<br>Diabetes May 2015;64(5):1760-9, 2015 May	2015	To investigate whether hypoglycemia awareness and unawareness are associated with different hypoglycemia-associated EEG changes
Selam JL.; Medlej R.; M'bemba J.; Chevalier A.; Guyon F.; Ashworth L.; Slama G.<br>Symptoms, hormones, and glucose fluxes during a gradual hypoglycaemia induced by intraperitoneal vs venous insulin infusion in Type I diabetes.<br>Diabetic medicine : a journal of the British Diabetic Association Dec 1995;12(12):1102-9, 1995 Dec	1995	To assess the indirect effects of IP insulin via lower insulinaemia
Shapiro ET.; Cooper M.; Chen CT.; Given BD.; Polonsky KS.<br>Change in hexose distribution volume and fractional utilization of [18F]-2-deoxy-2-fluoro-D-glucose in brain during acute hypoglycemia in humans.<br>Diabetes Feb 1990;39(2):175-80, 1990 Feb	1990	We used positron emission tomography (PET) to study the effects of mild hypoglycemia on cerebral glucose uptake and metabolism
Sherwin RS.<br>Evaluation of hypoglycemic counterregulation using a modification of the Andres glucose clamp.<br>Experimental gerontology ;28(4-5):371-80	1993	To study counterregulatory mechanisms against hypoglycemia
Shilo S.; Shamoon H.<br>Abnormal growth hormone responses to hypoglycemia and exercise in adults with type I diabetes.<br>Israel journal of medical sciences Mar 1990;26(3):136-41, 1990 Mar	1990	To investigate growth hormone response
Smith D.; Pernet A.; Reid H.; Bingham E.; Rosenthal JM.; Macdonald IA.; Umpleby AM.; Amiel SA.<br>The role of hepatic portal glucose sensing in modulating responses to hypoglycaemia in man.<br>Diabetologia Oct 2002;45(10):1416-24, 2002 Oct	2002	Investigated the effect of raising blood glucose concentrations in the hepatic-portal vein on neurohumoral

		responses during induction of systemic hypoglycaemia in nine healthy male volunteers
Siafarikas A.; Johnston RJ.; Bulsara MK.; O'Leary P.; Jones TW.; Davis EA.<br>Early loss of the glucagon response to hypoglycemia in adolescents with type 1 diabetes.<br>Diabetes care Aug 2012;35(8):1757-62, 2012 Aug	2012	To assess the glucagon response to hypoglycemia and identify influencing factors
Smith D.; Pernet A.; Rosenthal JM.; Bingham EM.; Reid H.; Macdonald IA.; Amiel SA.<br>The effect of modafinil on counter-regulatory and cognitive responses to hypoglycaemia.<br>Diabetologia Oct 2004;47(10):1704-11, 2004 Oct	2004	To assess if reducing release of the inhibitory neurotransmitter gamma-aminobutyric acid (GABA) with modafinil will enhance symptomatic and hormonal responses to hypoglycaemia
Snorgaard O.; Lassen LH.; Rosenfalck AM.; Binder C.<br>Glycaemic thresholds for hypoglycaemic symptoms, impairment of cognitive function, and release of counterregulatory hormones in subjects with functional hypoglycaemia.<br>Journal of internal medicine Apr 1991;229(4):343-50, 1991 Apr	1991	To investigate if subjects with functional hypoglycaemia may have inappropriately high glycaemic thresholds for the onset and perception of hypoglycaemia, as well as for the release of counterregulatory hormones
Sommerfield AJ.; Deary IJ.; McAulay V.; Frier BM.<br>Moderate hypoglycemia impairs multiple memory functions in healthy adults.<br>Neuropsychology Jan 2003;17(1):125-32, 2003 Jan	2003	To study the effects of acute insulin-induced hypoglycemia on short-term, delayed, and working
Sommerfield AJ.; Deary IJ.; McAulay V.; Frier BM.<br>Short-term, delayed, and working memory are impaired during hypoglycemia in individuals with type 1 diabetes.<br>Diabetes care Feb 2003;26(2):390-6, 2003 Feb	2003	To investigate the effects of experimentally induced hypoglycemia on verbal and nonverbal tests of short-term, delayed, and working memory
Soydan N.; Bretzel RG.; Fischer B.; Wagenlehner F.; Pilatz A.; Linn T.<br>Reduced capacity of heart rate regulation in response to mild hypoglycemia induced by glibenclamide and physical exercise in type 2 diabetes.<br>Metabolism: clinical and experimental May 2013;62(5):717-24, 2013 May	2013	To assess if HRV was impaired in mild hypoglycemia in diabetic individuals.
Spyer G.; Hattersley AT.; MacDonald IA.; Amiel S.; MacLeod KM.<br>Hypoglycaemic counter-regulation at normal blood glucose concentrations in patients with well controlled type-2 diabetes.<br>Lancet (London, England) Dec 2000;356(9246):1970-4, 2000 Dec	2000	To assess at which glucose concentrations symptoms and release of hormones takes place in patients with type 2 diabetes
Strachan MW.; Deary IJ.; Ewing FM.; Ferguson SS.; Young MJ.; Frier BM.<br>Acute hypoglycemia impairs the functioning of the central but not peripheral nervous system.<br>Physiology & behavior Jan 2001;72(1-2):83-92, 2001 Jan	2001	To assess the impact of hypoglycemia on the function of the peripheral nervous system.
Strachan MW.; Ewing FM.; Frier BM.; McCrimmon RJ.; Deary IJ.<br>Effects of acute hypoglycaemia on auditory information processing in adults with Type I diabetes.<br>Diabetologia Jan 2003;46(1):97-105, 2003 Jan	2003	To investigate acute hypoglycaemia in humans on general impairment of cognitive function

Steil GM.; Rebrin K.; Hariri F.; Jinagonda S.; Tadros S.; Darwin C.; Saad MF.<br>Interstitial fluid glucose dynamics during insulin-induced hypoglycaemia.<br>Diabetologia Sep 2005;48(9):1833-40, 2005 Sep	2005	To investigate interstitial fluid glucose dynamics during hypoglycaemia
Stevens AB.; McKane WR.; Bell PM.; Bell P.; King DJ.; Hayes JR.<br>Psychomotor performance and counterregulatory responses during mild hypoglycemia in healthy volunteers.<br>Diabetes care Jan 1989;12(1):12-7, 1989 Jan	1989	To study psychomotor performance and counterregulatory responses during mild hypoglycemia in healthy volunteers
Taverna MJ.; M'Bemba J.; Sola A.; Chevalier A.; Slama G.; Selam JL.<br>Insufficient adaptation of hypoglycaemic threshold for cognitive impairment in tightly controlled type 1 diabetes.<br>Diabetes & metabolism Feb 2000;26(1):58-64, 2000 Feb	2000	To investigate the threshold for cognitive impairment
ter Braak EW.; Appelman AM.; van der Tweel I.; Erkelens DW.; van Haeften TW.<br>The sulfonylurea glyburide induces impairment of glucagon and growth hormone responses during mild insulin-induced hypoglycemia.<br>Diabetes care Jan 2002;25(1):107-12, 2002 Jan	2002	Investigating the impact of glyburide on glucose counterregulatory hormones during stepwise hypoglycemic clamp studies
Terpstra M.; Moheet A.; Kumar A.; Eberly LE.; Seaquist E.; Öz G.<br>Changes in human brain glutamate concentration during hypoglycemia: insights into cerebral adaptations in hypoglycemia-associated autonomic failure in type 1 diabetes.<br>Journal of cerebral blood flow and metabolism : official journal of the International Society of Cerebral Blood Flow and Metabolism May 2014;34(5):876-82, 2014 May	2014	To follow the human brain glutamate concentration during experimentally induced hypoglycemia in subjects with and without HAAF
Towler DA.; Havlin CE.; Craft S.; Cryer P.<br>Mechanism of awareness of hypoglycemia. Perception of neurogenic (predominantly cholinergic) rather than neuroglycopenic symptoms.<br>Diabetes Dec 1993;42(12):1791-8, 1993 Dec	1993	To determine which symptoms of hypoglycemia are reproducible, 2) to pharmacologically distinguish neurogenic (autonomic) from neuroglycopenic symptoms, and 3) to test the hypothesis that awareness of hypoglycemia is the result of perception of neurogenic rather than neuroglycopenic symptoms.
Trajanoski Z.; Brunner GA.; Schaupp L.; Ellmerer M.; Wach P.; Pieber TR.; Kotanko P.; Skrabal F.<br>Open-flow microperfusion of subcutaneous adipose tissue for on-line continuous ex vivo measurement of glucose concentration.<br>Diabetes care Jul 1997;20(7):1114-21, 1997 Jul	1997	To evaluate a novel technique for on-line continuous glucose measurement in subcut aneous adipose tissue, and to investigate its accuracy for detection of hypoglycemia
Tschritter O.; Schäfer SA.; Klett J.; Pfäfflin A.; Häring HU.; Hennige AM.; Fritsche A.<br>Insulin detemir causes increased symptom awareness during hypoglycaemia compared to human insulin.<br>Diabetes, obesity & metabolism Nov 2009;11(11):1017-26 2009 Nov	2009	To test whether insulin detemir leads to altered hormone and symptom response during hypoglycaemia
Tsui EY.; Chiasson JL.; Tildesley H.; Barnie A.; Simkins S.; Strack T.; Zinman B.<br>Counterregulatory hormone responses after long-term continuous subcutaneous insulin infusion with lispro insulin.<br>Diabetes care Jan 1998;21(1):93-6 1998 Jan	1998	To determine whether the long-term use of insulin lispro (LP) affects the counterregulatory h

		ormone response to hypoglycemia
van de Ven KC.; van der Graaf M.; Tack CJ.; Klomp DW.; Heerschap A.; de Galan BE.<br>Optimized [1-(13)C]glucose infusion protocol for 13C magnetic resonance spectroscopy at 3T of human brain glucose metabolism under euglycemic and hypoglycemic conditions.<br>Journal of neuroscience methods Jan 2010;186(1):68-71, 2010 Jan	2010	To test the feasibility to study human brain glucose metabolism
van de Ven KC.; de Galan BE.; van der Graaf M.; Shestov AA.; Henry PG.; Tack CJ.; Heerschap A.<br>Effect of acute hypoglycemia on human cerebral glucose metabolism measured by ^{13}C magnetic resonance spectroscopy.<br>Diabetes May 2011;60(5):1467-73, 2011 May	2011	To investigate the effect of acute insulin-induced hypoglycemia on cerebral glucose metabolism in
van de Ven KC.; Tack CJ.; Heerschap A.; van der Graaf M.; de Galan BE.<br>Patients with type 1 diabetes exhibit altered cerebral metabolism during hypoglycemia.<br>The Journal of clinical investigation Feb 2013;123(2):623-9, 2013 Feb	2013	To assess the metabolic effects of hypoglycemia in patients with T1DM
Vea H.; Jorde R.; Sager G.; Vaaler S.; Sundsfjord J.<br>The effect of selective beta 1-blockade on glucose thresholds for release of counterregulatory hormones and symptoms in insulin-dependent diabetes mellitus.<br>Acta endocrinologica Mar 1992;126(3):264-70, 1992 Mar	1992	To examine whether plasma glucose thresholds for counterregulatory hormones and symptoms are influenced by the beta1-selective blocker metoprolol
Vea H.; Jorde R.; Sager G.; Vaaler S.; Sundsfjord J.<br>Reproducibility of glycaemic thresholds for activation of counterregulatory hormones and hypoglycaemic symptoms in healthy subjects.<br>Diabetologia Oct 1992;35(10):958-61, 1992 Oct	1992	To look at the reproducibility of hypoglycaemic thresholds for activation of neuroendocrine responses and symptoms
Vea H.; Jorde R.; Sager G.; Vaaler S.; Sundsfjord J.<br>Glycemic thresholds for hypoglycemic responses in obese subjects.<br>International journal of obesity and related metabolic disorders : journal of the International Association for the Study of Obesity Feb 1994;18(2):111-6, 1994 Feb	1994	To compare glycaemic treshholds for hypoglycaemia in obese and control subjects
Verdonk CA.; Rizza RA.; Westland RE.; Nelson RL.; Gerich JE.; Service FJ.<br>Glucose clamping using the Biostator GCIIS.<br>Hormone and metabolic research = Hormon- und Stoffwechselforschung = Hormones et metabolisme Apr 1980;12(4):133-5, 1980 Apr	1980	To assess the feasibility of utilizing the Biostator GCIIS in maintaining glucose clamps.
Verdonk CA.; Rizza RA.; Nelson RL.; Go VL.; Gerich JE.; Service FJ.<br>Interaction of fat-stimulated gastric inhibitory polypeptide on pancreatic alpha and beta cell function.<br>The Journal of clinical investigation May 1980;65(5):1119-25, 1980 May	1980	the effects of differing blood glucose levels on the insulinotropic and glucagonotropic actions of fat-stimulated gastric inhibitory polypeptide
Wahren J .; Ekberg K.; Fernqvist-Forbes E.; Nair S.<br>Brain substrate utilisation during acute hypoglycaemia.<br>Diabetologia Jul 1999;42(7):812-8, 1999 Jul	1999	To determine the utilisation of glucose and alternative substrates, in particular amino acids, during hypoglycaemia
Ward DS.; Voter WA.; Karan S.<br>The effects of hypo- and hyperglycaemia on the hypoxic ventilatory response in humans.<br>The Journal of physiology Jul 2007;582(Pt 2):859-69, 2007 Jul	2007	To determine whether hypo- and hyperglycaemia modulate the hypoxic ventilatory response

Warren RE.; Zammitt NN.; Deary IJ.; Frier BM.<br>The effects of acute hypoglycaemia on memory acquisition and recall and prospective memory in type 1 diabetes.<br>Diabetologia Jan 2007;50(1):178-85, 2007 Jan	2007	The effects of acute hypoglycaemia on different memory processes
Warren RE.; Sommerfield AJ.; Greve A.; Allen KV.; Deary IJ.; Frier BM.<br>Moderate hypoglycaemia after learning does not affect memory consolidation and brain activation during recognition in non-diabetic adults.<br>Diabetes/metabolism research and reviews ;24(3):247-5	2008	Determine if a period of hypoglycaemia occurring immediately after exposure to new stimuli impaired subsequent recognition
Watson JM.; Sherwin RS.; Deary IJ.; Scott L.; Kerr D.<br>Dissociation of augmented physiological, hormonal and cognitive responses to hypoglycaemia with sustained caffeine use.<br>Clinical science (London, England : 1979) Apr 2003;104(4):447-54<br>2003 Apr	2003	To determine whether symptomatic and counterregulatory responses are lost with sustained caffeine use, i.e. does tolerance develop
Weinger K.; Jacobson AM.; Draelos MT.; Finkelstein DM.; Simonson DC.<br>Blood glucose estimation and symptoms during hyperglycemia and hypoglycemia in patients with insulin-dependent diabetes mellitus.<br>The American journal of medicine Jan 1995;98(1):22-31, 1995 Jan	1995	Determine hypoglycemic and hyperglycemic symptoms, accuracy of estimating blood glucose, and their relation to glycemic control and counterregulatory hormone levels
Welt CK.; Kinsley BT.; Simonson DC.<br>Recurrent hypoglycemia does not impair the cortisol response to adrenocorticotropin infusion in healthy humans.<br>Metabolism: clinical and experimental Oct 1998;47(10):1252-7, 1998 Oct	1998	Determine the effect of corticotropin infusion after antecedent hypogylcaemia on the cortisol response
Wellhoener P.; Fruehwald-Schultes B.; Kern W.; Dantz D.; Kerner W.; Born J.; Fehm HL.; Peters A.<br>Glucose metabolism rather than insulin is a main determinant of leptin secretion in humans.<br>The Journal of clinical endocrinology and metabolism Mar 2000;85(3):1267-71, 2000 Mar	2000	The effect of glucose uptake on leptin secretion
Wiegers EC.; Rooijackers HM.; Tack CJ.; Heerschap A.; de Galan BE.; van der Graaf M.<br>Brain Lactate Concentration Falls in Response to Hypoglycemia in Patients With Type 1 Diabetes and Impaired Awareness of Hypoglycemia.<br>Diabetes 06 2016;65(6):1601-5, 2016 06.	2016	Determine the effect of acute hypoglycemia on brain lactate concentration
Widom B.; Simonson DC.<br>Intermittent hypoglycemia impairs glucose counterregulation.<br>Diabetes Dec 1992;41(12):1597-602, 1992 Dec	1992	To determine whether intermittent hypoglycemia would attenuate the autonomic and/or neuroglycopenic symptoms associated with low circulating glucose levels.
Woerle HJ.; Meyer C.; Popa EM.; Cryer PE.; Gerich JE.<br>Renal compensation for impaired hepatic glucose release during hypoglycemia in type 2 diabetes: further evidence for hepatorenal reciprocity.<br>Diabetes Jun 2003;52(6):1386-92, 2003 Jun	2003	To determine the hepatic and renal glucose release during hypoglyaemia
Wright RJ.; Frier BM.; Deary IJ.<br>Effects of acute insulin-induced hypoglycemia on spatial abilities in adults with type 1 diabetes.	2009	Determine the effects of hypoglycaemia on spatial cognitive abilitie

Diabetes care Aug 2009;32(8):1503-6, 2009 Aug		
Zammitt NN.; Warren RE.; Deary IJ.; Frier BM.<br>Delayed recovery of cognitive function following hypoglycemia in adults with type 1 diabetes: effect of impaired awareness of hypoglycemia.<br>Diabetes Mar 2008;57(3):732-6, 2008 Mar	2008	Determine the recovery of cognitive function after hypoglycaemia
Zenz S, Mader JK, Regittnig W, et al. Impact of C-Peptide Status on the Response of Glucagon and Endogenous Glucose Production to Induced Hypoglycemia in T1DM. *J Clin Endocrinol Metab*. 2018;103(4):1408-1417. doi:10.1210/jc.2017-01836	2018	Determine the impact of C-peptide status on glucagon response and endogenous glucose production (EGP) during hypoglycemia
Zijlstra E.; Heinemann L.; Fischer A.; Kapitza C.<br>A Comprehensive Performance Evaluation of Five Blood Glucose Systems in the Hypo-, Eu-, and Hyperglycemic Range.<br>Journal of diabetes science and technology 11 2016;10(6):1316-1323 2016 11	2016	Evaluate the performance of 5 CE-certified and commercially available blood glucose (BG) systems

ESM Table 2: Dietary instructions prior to the clamp

Dietary instructions	*Number (%)*
No dietary instruction	57 (14.9)
Overnight fast	310 (80.9)
Weight maintaining diet	40 (10.4)
Standardised meal evening prior to the clamp	12 (3.1)
Meals containing 200 carbs	10 (2.6)
Standard breakfast	8 (2.1)
Isocaloric meals	6 (1.6)
Regular meals	4 (1.0)
Unspecified	2 (0.5)

The numbers add up to >100%, as the dietary instructions are not mutually exclusive.

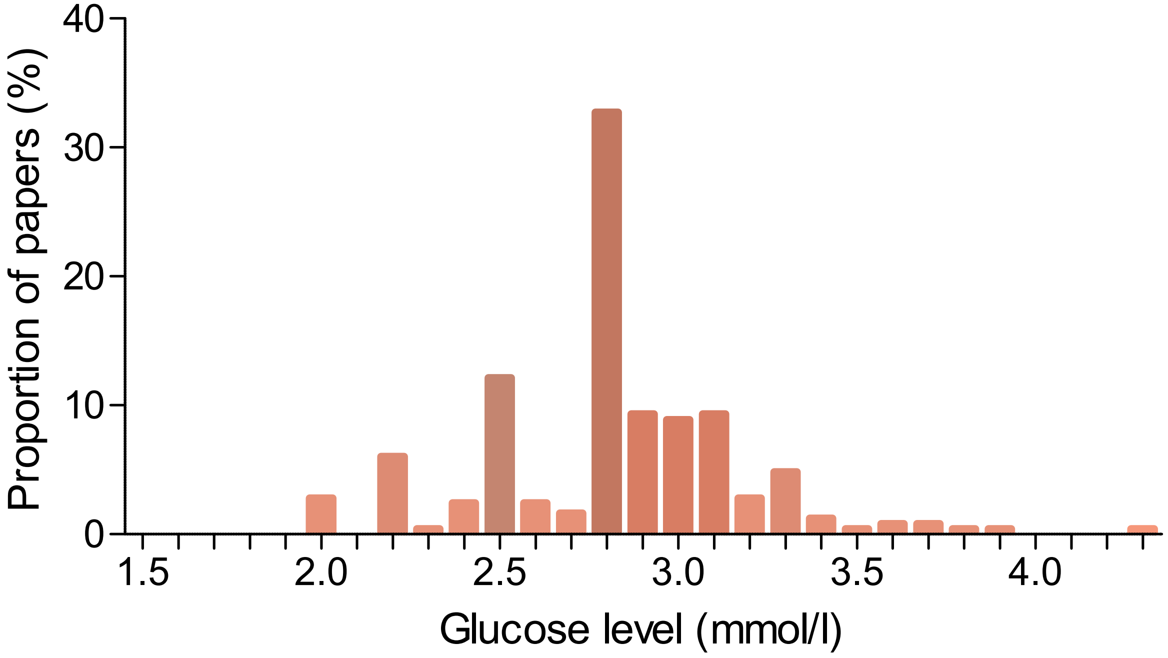

ESM Fig.1 Hypoglycaemic nadir in single-step clamps

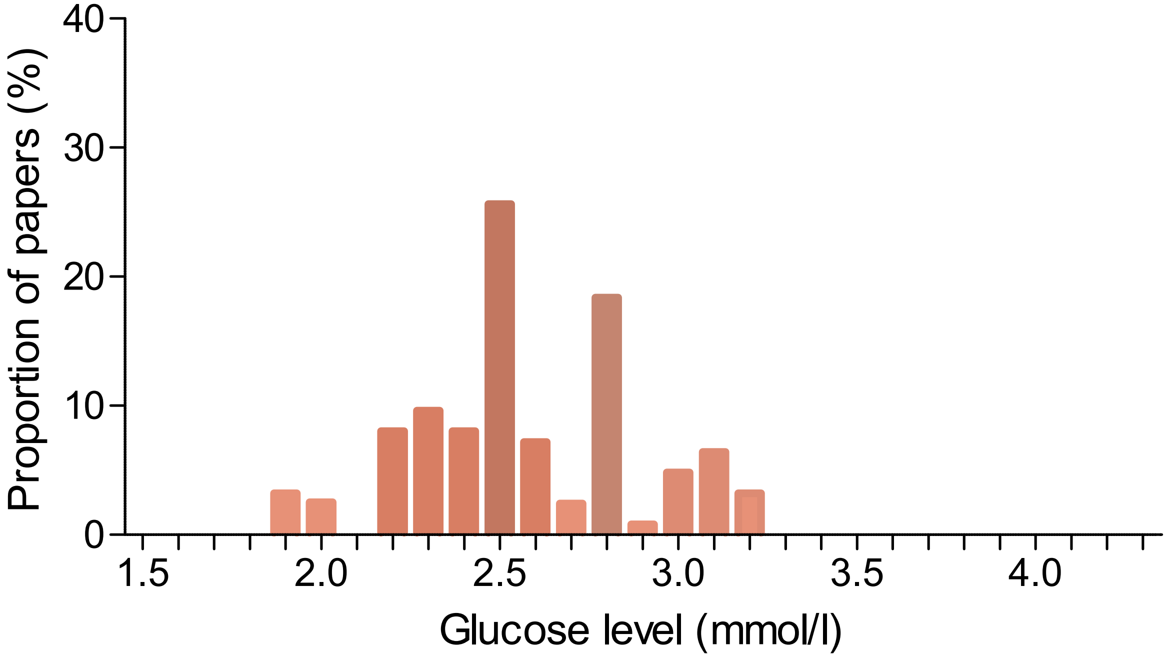

ESM Fig.2 Hypoglycaemic nadir in multiple stepped clamps
